# Generating Long-Lived Highly Excited Carriers in Undoped Perovskite Nanostructures by Two-Photon Upconversion

**DOI:** 10.1021/acs.nanolett.6c02392

**Published:** 2026-07-30

**Authors:** Hackim Musah, Thilina Gamagedara, Estehad Pathan, Amos Afugu, Atish Ghosh, Suk Kyoung Lee, Zhen-Fei Liu, Aaron S. Rury, Wen Li

**Affiliations:** Department of Chemistry, 2954 Wayne State University, Detroit, Michigan 48202, United States

**Keywords:** photovoltaics, photoinduced thermionic emission, perovskite nanoplatelets, electron momentum imaging, long-lived carriers

## Abstract

Delayed electron emissions from undoped metal halide perovskite (CsPbBr_3_) nanostructures upon laser irradiation were observed to persist for a few nanoseconds. This was attributed to an extended lifetime of upconverted highly excited carriers. Experimental evidence suggests that the excitation of such states is through one-photon absorption by carriers and excitons. Theoretical work reveals that surface defects play an important role in modifying the band edge energies of the nanoparticles and thus allow the presence of these highly excited carrier states.

## Graphical abstract

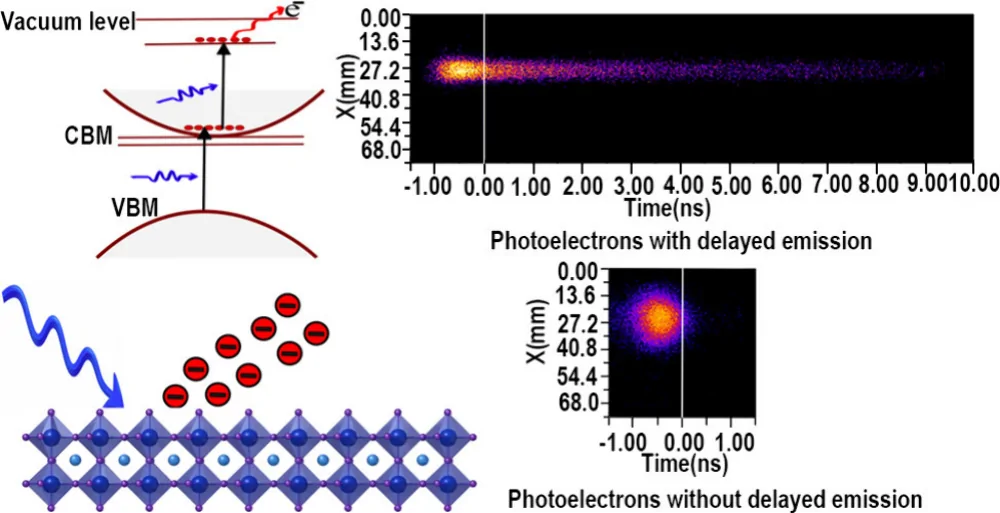

Halide perovskites have emerged as an important class of materials in optoelectronics, owing to their outstanding properties. Integrating these materials into hot-carrier solar cells (HCSCs) offers a promising route to exceed the Shockley–Queisser efficiency limit by enabling energy-selective extraction of photocarriers before full thermalization.^1,2^ Unlike conventional photovoltaic devices, which harvest carriers only after they have relaxed to the band edge, HCSCs aim to extract charge carriers while they remain in high-energy, nonequilibrium (“hot”) states,^3,4^ thereby enabling more efficient solar energy conversion through hot carrier extraction mechanisms.^5–7^ However, this is quite challenging because of the extremely short lifetime of hot carriers, even in perovskite material (less than a few ps).^8–12^

Prior work has shown that halide perovskite nanocrystals can generate highly excited carriers through two-photon upconversion.^13,14^ For example, in manganese-doped CsPbBr_3_ systems, exploiting the extremely long lifetime of Mn^2+^ excited states (tens of μs), two-photon upconverted highly excited carriers can be produced. The energy of these carriers is so high that thermionic emission from these states becomes possible at room temperature, which was used to infer the existence of these highly excited carriers. However, despite intensive studies on such a scheme in the past decade, some important questions remain unanswered: (1) What is the lifetime of these highly excited carriers? (2) Can the up-conversion scheme be implemented without a dopant? and (3) if yes, what is the mechanism by which these highly excited carriers are populated?

While a variety of advanced time-resolved spectroscopic methods have been used to probe the carrier lifetime in perovskites in general, such as transient absorption spectroscopy and microscopy,^15–18^ and time-resolved angular-resolved photoemission spectroscopy (tr-ArPES),^19–22^ there has been no report to date on the lifetime of the highly excited carriers. Recent studies employing the 3D momentum imaging system, developed by Li and co-workers, have investigated hot-carrier decay dynamics in graphene,^23^ laser desorption ionization behavior of 2,5-dihydroxybenzoic acid (DHB),^24^ and photoelectrons arising from photoemission of 2,5-DHB.^25^ The 3D momentum technique employs a “pump and watch” approach, which can measure the lifetime with a single laser beam. The momentum imaging system offers a means of capturing both the momentum distribution and temporal characteristics of photoemitted electrons. This allows one to directly measure the lifetime of hot carriers directly without a pump–probe setup.^23^ Here, in this work, we extend this methodology to determine the lifetime of highly excited carriers excited by two-photon upconversion in a species of lead halide nanoparticles by imaging the momentum of emitted electrons and show that the lifetime of these carriers is surprisingly long at a few nanoseconds. We also showed that the highly excited carriers can be produced in undoped metal halide perovskites in both nanoplatelet and nanocrystal form, but not in bulk. We examine different potential excitation mechanisms capable of explaining our experimental and computational findings.

The experimental setup, comprising a vacuum chamber and a velocity map imaging (VMI) spectrometer, follows a configuration for 2*D*/3D VMI, as detailed in previous studies.^23,25^ Picosecond and femtosecond laser pulses were incorporated into the system to generate three-dimensional momentum-resolved photoelectron images from various thin film samples. The second (532 nm), third (355 nm), and fifth (213 nm) harmonics were generated from a Nd:YAG picosecond laser system operating at a fundamental wavelength of 1064 nm, characterized by a pulse duration of approximately 10 ps and a repetition rate of 1 kHz. The 213 nm fifth harmonic was obtained via sum-frequency mixing of the 355 and 532 nm photons in a β-BaB_2_O_4_ (BBO) nonlinear crystal. In addition, the fundamental wavelength (775 nm), along with its second (382 nm) and third (255 nm) harmonics, was generated using a Ti:sapphire laser system (KMLabs, Red Dragon, 30 fs) operating at 1 kHz. The 382 nm photon was produced via second-harmonic generation (SHG) of the 775 nm fundamental in a BBO crystal. Subsequently, the 255 nm ultraviolet beam was generated through sum-frequency generation (SFG) of the 775 and 382 nm beams in a separate BBO crystal. The exact wavelengths were confirmed with a spectrometer.

Two-monolayer (2 ML) CsPbBr_3_ nanoplatelets (NPLs) with face-down and edge-up orientations were prepared following established procedures, which are detailed in the SI.^26^ Colloidal dispersions were drop-cast onto Si wafers and allowed to dry under ambient conditions. The substrate-mounted films were secured to a stainless-steel ring using conductive Cu tape and installed on the repeller electrode of the velocity-map imaging apparatus inside a vacuum chamber (base pressure ∼10^–9^ Torr) at room temperature, with the film facing the laser beam and the detector. Bulk CsPbBr_3_ (Nanochemazone) was processed into thin films onto ITO-coated substrates as described elsewhere^27^ and measured under identical conditions. CsPbBr_3_ quantum dots (QDs) (Sigma-Aldrich) were used as received and deposited by drop-casting onto ITO-coated substrates. Photoelectrons were collected from all samples with the velocity-map imaging spectrometer under the same operating conditions as described above.

Figure 1 presents the structural and optical characterization of the 2 ML CsPbBr_3_ nanoplatelets prepared from hexane and toluene dispersions. Representative HAADF-STEM images are shown in Figure 1a,b. The hexane-cast sample shows uniformly dispersed, square-shaped nanoplatelets with lateral dimensions of approximately 30 nm, whereas the toluene-cast sample appears as aligned ribbon-like structures. These features appear because the CsPbBr_3_ nanoplatelets arrange with one lateral dimension perpendicular to the Si substrate, making them appear as linear structures that form larger ribbon-like assemblies. Figure 1c shows the absorption spectra of the 2 ML CsPbBr_3_ nanoplatelets dispersed in hexane and toluene as a function of photon energy. Both spectra exhibit a single, prominent band-edge absorption peak at approximately 430 nm, corresponding to 2.88 eV, indicating that the same 2 ML CsPbBr_3_ nanoplatelet phase is formed in both dispersion samples. Vertical lines indicate the photon energies corresponding to the excitation wavelengths used in this study, allowing direct comparison between the pump energies and the band-edge absorption.

**Figure 1:**
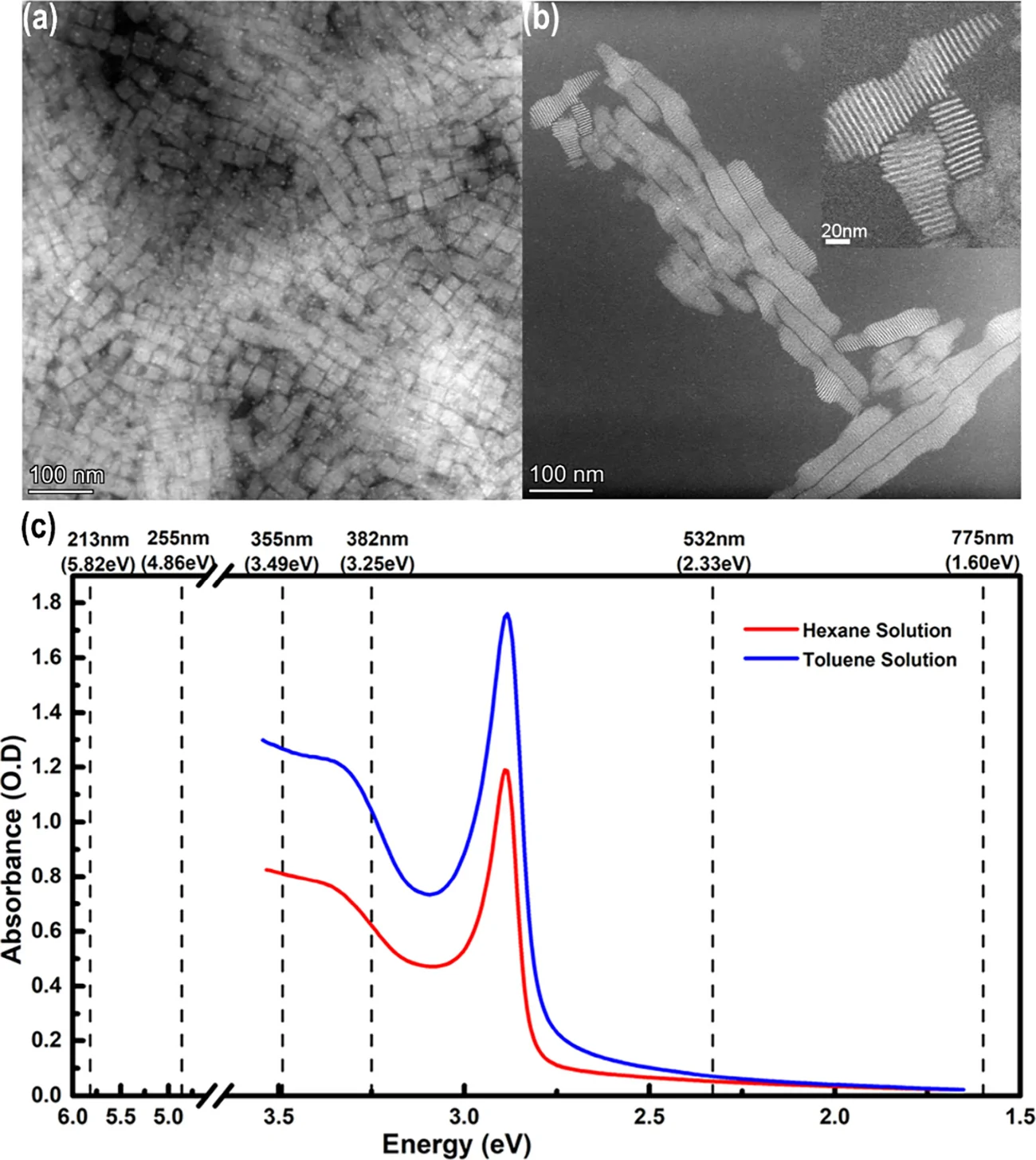
Structural and optical characterization of 2 ML CsPbBr_3_ nanoplatelets. Representative HAADF-STEM images of nanoplatelet films prepared from (a) hexane and (b) toluene dispersions. (c) Absorption spectra of 2 ML CsPbBr_3_ nanoplatelets dispersed in hexane and toluene, plotted as a function of photon energy. Vertical dashed lines indicate the photon energies corresponding to the excitation wavelengths used in this study.

Figure 2a illustrates the experimental setup. The laser pulse excites the sample film, and the emitted photoelectrons are accelerated by an electric field provided by VMI electrodes (L1-L5) toward the detector. The position where the electron impacts the XY-plane of the detector encodes the parallel momenta of the emitted electrons, while the time-of-flight (TOF,t) reflects their momenta normal to the sample surface. Figure 2b–g show spatial–TOF (X-t) reconstructed three-dimensional photoelectron Newton spheres from 2-ML CsPbBr_3_ nanoplatelets excited at 355, 382, 532, 775, 213, and 255 nm. Y-t distributions are similar (not shown). The vertical white line marks the TOF time zero, t_0_, defined as the TOF of electrons with zero momentum along the TOF axis; electrons with a longitudinal momentum component toward the detector arrive earlier than t_0_ (negative TOF). For 355 and 382 nm excitation (Figure 2b,c), the distributions display a substantial fraction of electrons arriving after t_0_ (positive time) that extends up to 8–10 ns; this delayed component dominates the integrated signal. In contrast, at 532, 775, 213, and 255 nm (Figure 2d–g), emission is predominantly prompt with only a weak tail that decays within ≲0.25 ns, i.e., no appreciable delayed component appears within the measurement window. Here, we attribute the delayed emission (at 382 and 355 nm) to thermionic-like emission of highly excited carriers, and the decay time reflects the lifetime of these carriers. This is similar to those observed in graphene.^23^ Following the phenomenological picture, with the measured count rate, we can estimate the work function of such states with the Richardson-Dushman equation,^28^ $J=AT^{2}e^{-\phi /k_{B}T}$ , which assumes the lattice temperature to be the room temperature with the Richardson constant as 1.79 × 10^5^ A/m^2^K^2^.^29^ The resulting work function is about 0.9 eV. Considering the affinity energy of perovskite nanoplatelet is ∼4 eV,^30^ this would put the excess energy of these carrier states ∼3.1 eV above the conduction band minimum. In Figure 3a, we examine the decay kinetics of the delayed emission, which shows the post *t*_0_ electron-yield transients, with all traces normalized to unity. The 532 and 775 nm data decay rapidly to the background level with no resolvable long tail, consistent with the absence of delayed emission in Figure 2d,e. Excitation at 213 and 255 nm exhibits similar behavior. Because all four wavelengths show indistinguishable dynamics after *t*_0_, only the 532 and 775 nm traces are plotted as representatives. By contrast, the 382 and 355 nm traces decay more slowly and retain substantial signal well past *t*_0_.

**Figure 2:**
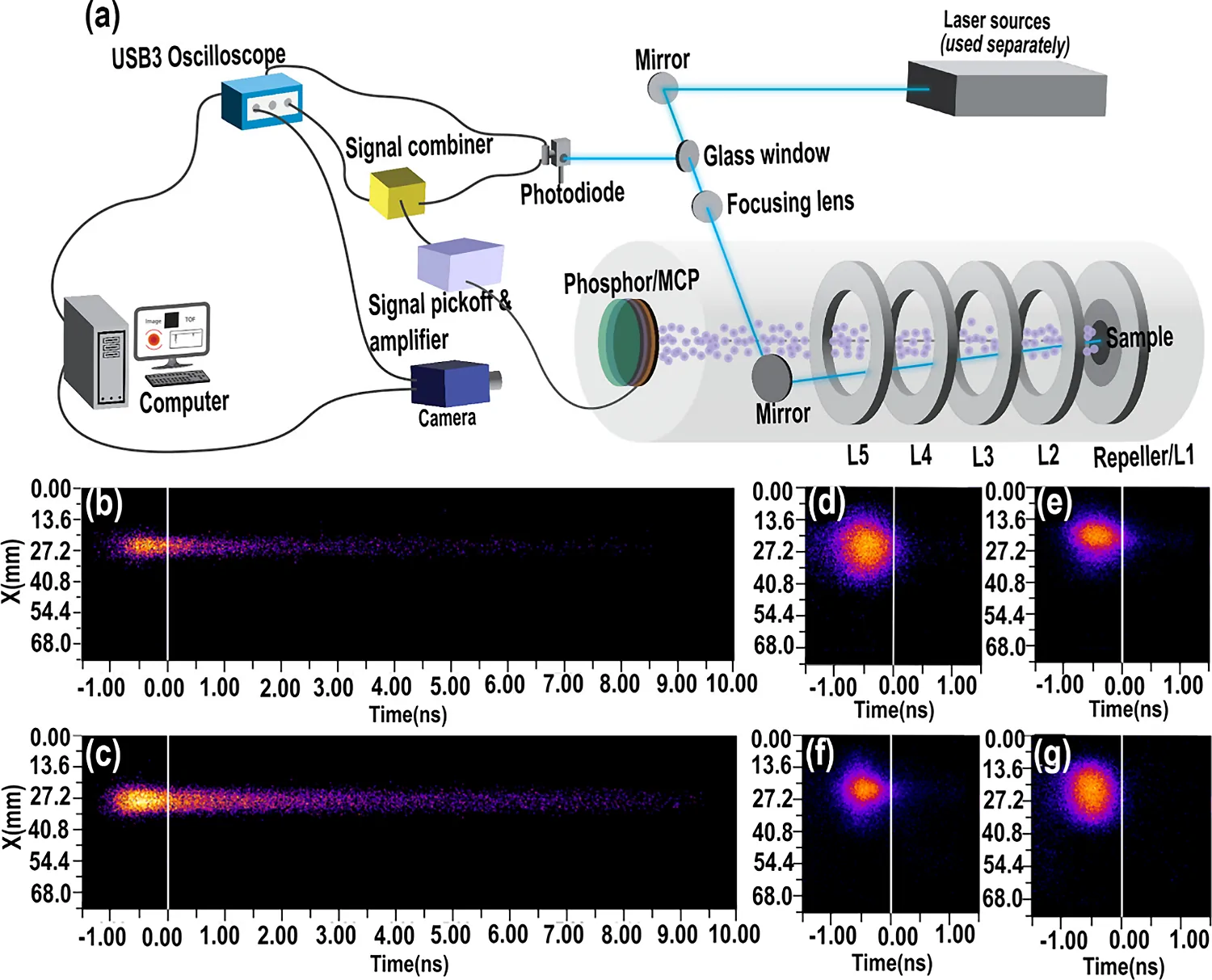
(a) Schematic of the experimental setup. (b–g) Spatial-TOF distributions of photoelectrons emitted from face-down 2-ML CsPbBr_3_ nanoplatelets. The horizontal axis is the TOF axis while X is a transverse spatial coordinate. Time zero (p_*z*_ = 0), as specified in the main text, is indicated by the vertical white line. Excitation wavelengths: (b) 355 nm, (c) 382 nm, (d) 532 nm, (e) 775 nm, (f) 213 nm, and (g) 255 nm.

**Figure 3:**
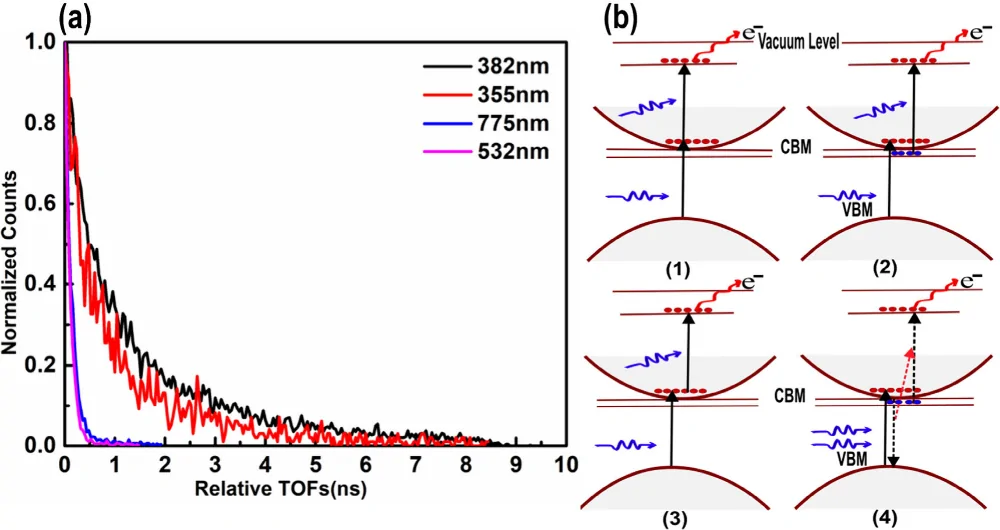
(a) Photoelectron TOF decay curves after time zero under 355, 382, 775, and 532 nm excitation. (b) Schematic illustration of four possible photophysical pathways for highly excited carrier upconversion. See main text for a description of each pathway.

To quantify these trends, we fit the normalized transients with exponential models. A single exponential adequately describes the 532 and 775 nm curves, yielding decay constants of 113.9 and 132.7 ps, respectively, which we assign as the falling edge of the instrument response function (IRF) for TOF measurement. The 382 and 355 nm data require biexponential forms, revealing both fast and slow components. For 382 nm, we obtain τ_fast_ = 512.2 ps and τ_slow_ = 2.159 ns, for 355 nm, τ_fast_ = 312.0 ps and τ_slow_ = 1.541 ns. This confirms that the lifetime of these highly excited carriers can reach up to more than 2 ns. As far as we know, this is the first time that such states and their lifetime were characterized experimentally. The fast decay component is significantly slower than the TOF IRF and the conventional edge carrier cooling time^8–12^ and suggests that they represent intrinsic decay dynamics. We tentatively assign the fast component to relaxation or redistribution of the initial higher-energy population as it evolves toward the longer-lived highly excited states.

We note these states might be the same carrier state accessed in manganese-doped perovskite platelets from previous studies.^14^ However, because our sample has no manganese dopants, the excitation mechanism will differ from previous studies. We consider the following four different processes: (1) Direct multiphoton excitation, where two-photon transitions (355 and 382 nm) directly populate the highly excited carriers. (2) Photoexcitation of excitons, after initially cooling photoexcited hot carriers. (3) Photoexcitation of hot carriers, before cooling. (4) Auger recombination between two excitons after cooling. Mechanism 1 is disputed by the fact that the delayed emission can only be produced by 382 and 355 nm, but not by 775 or 532 nm photons. This is because three 532 nm photons have the same energy as two 355 nm photons, while that of four 775 nm photons is close to two 382 nm photons. The selection rule might suggest two- and three-photon transitions access different states due to symmetry, even though their energy is the same. However, for 382 and 775 nm, both need an even number of photons and thus likely share the same excited states if direct photoexcitation is the correct mechanism. This is not what was observed: even though photoemission was detected at a higher laser fluence (See Table S2 in Supporting Information for data), 775 nm excitation produces no delayed emission. Therefore, mechanism 1 can be ruled out. Furthermore, double logarithm plots between laser fluence and photoelectron count rates showed a slope less than 2 for both 382 and 355 nm (see Figure S7 in Supporting Information), which suggests a two-photon processes mediated by a resonance step. In mechanism 2, one photon is first absorbed to produce a hot carrier, whose energy reduces upon exciton formation; a second photon is then absorbed by the exciton, and a highly excited carrier is produced. This process involves the cooling of a hot carrier and is strongly dependent on the relationship between the laser pulse duration and carrier cooling lifetime. The effective 382 nm excitation pulse duration at the sample position was experimentally measured to be ∼145 fs and the hot carrier cooling time was measured to be ∼2 ps using two photon photoemission (2PPE) (Figure S8 and discussion in Supporting Information). Therefore, a substantial fraction of the hot-carrier population remains when the second photon is absorbed, making Mechanism 3 more likely under 382 nm excitation. We note that hot carrier lifetimes of around 70 to 140 fs have been reported for colloidal perovskite nanoplatelets;^8,12,31^ however, the stacked nanoplatelet “superlattice” on substrates can increase the cooling time significantly, due to the hot-phonon bottleneck effect.^32,33^ Our measured value is consistent with these observations where the carrier cooling time becomes longer than for the case of solvated individual NPLs. For 355 nm, mechanism 2 is operable because the pulse duration is long at 10 ps. Mechanism 4 involves a biexciton decay and therefore should not depend on the excitation wavelengths or pulse duration, if the laser fluence is high enough. The observed wavelength sensitivity renders this mechanism nonoperable. Below, we provide further evidence to support our determination of the mechanisms at different laser wavelengths.

We observe interesting behavior in the parallel momentum of emitted electrons, as shown in Figure 4a,b. The size of the XY image reflects the parallel momentum of the emitted electrons. We can integrate and convert the momentum distributions to energy distributions (Figure 4c). It appears that the electron energy arising from 355 nm irradiation is less than that from 382 nm. This is somewhat surprising because the total energy of two 355 nm photons is higher than that of 382 nm (6.98 eV vs 6.49 eV), and one would expect the emitted electrons should have a higher energy. However, once we take into account the pulse duration difference between 355 nm (10 ps) and 382 nm (140 fs) and the difference between mechanism 2 and 3, it becomes apparent that the hot carrier cooling play an important role: using the excitonic band gap of the 2-ML CsPbBr_3_ nanoplatelets (E_g_≈ 2.88 eV), estimated from the absorption peak near 430 nm in Figure 1c, an energy loss of 0.61 eV is incurred for 355 nm excitation due to cooling and this puts the total energy of the highly excited carrier (electrons) to be about 3.49 eV above the exciton energy. Note this value is consistent with that obtained from Richard-Dunshman eq (3.1 eV) when using an exciton binding energy of ∼0.3 eV.^34^ While for 382 nm, the total energy is 3.61 eV above the exciton energy, which is slightly higher than that with 355 nm excitation. This would nicely explain the difference in the momentum distribution: the slightly lower energy needed to reach the vacuum level with the 382 nm excitation will allow electrons with a broader parallel momentum to have enough perpendicular energy to be emitted. Another observation also supports the existence of the highly excited carrier states and the proposed mechanism: the absence of delayed photoemission with excitation wavelengths of 255 nm (4.86 eV) and 213 nm (5.82 eV). These photon energies are too high to further excite the excitons/carriers. Instead, they will simply ionize them because the electron affinity is ∼4.0 eV.

**Figure 4:**
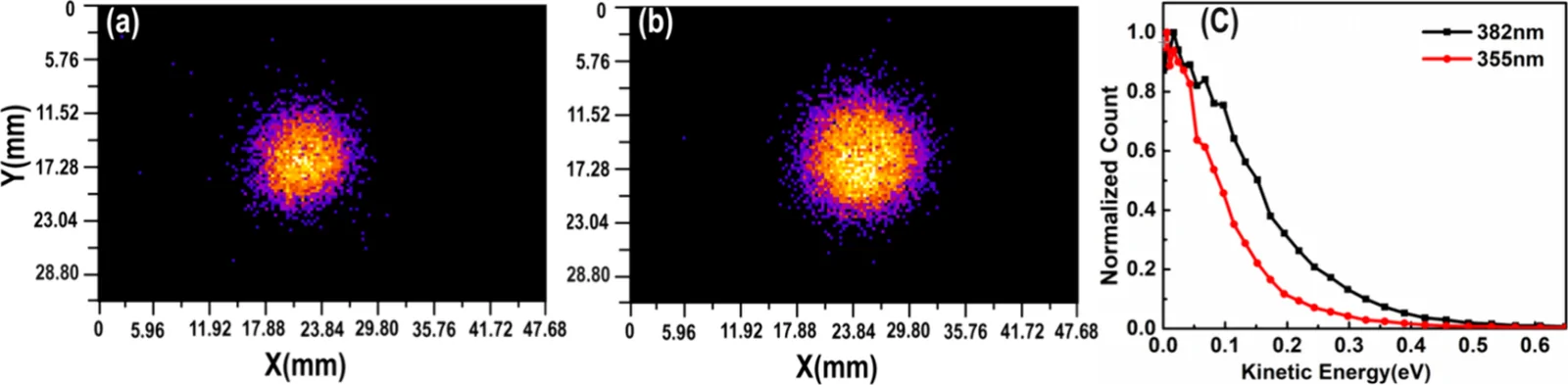
Parallel momentum distributions of delay emitted photoelectrons represented by the XY image size under excitation by (a) 355 nm and (b) 382 nm. (c) Kinetic energy distributions extracted from panels a and b.

Measurements were also carried out for edge-up oriented 2 ML NPLs. These samples serve as important controls to better understand the physical mechanisms leading to the observed thermionic emission in the face-down samples. For the edge-up oriented 2 ML NPLs, the surface exposed to vacuum conditions in our experiment is not passivated by organic ligands. If we observe similar delayed photoemission, then we know that the photoinduced thermionic emission is not due to organic ligand effect and an uncovered plane of the inorganic sublattice can also absorb incident photons.

Indeed, the wavelength dependence of delayed emission from the edge-up samples is qualitatively similar as in Figure 2b–g: mixed prompt-and-delayed emission at 355 and 382 nm, and predominantly prompt dynamics at 532, 775, 213, and 255 nm, indicating that the delayed component is intrinsic and largely insensitive to film orientation (please see Figure S5 in Supporting Information). Fluence-dependent measurements were performed to assess whether excitation density alters the TOF decay of the photoelectron signal. Across the accessible fluence range, the extracted decay constants showed no statistically significant variation, indicating that, under our conditions, the delayed component is not strongly influenced by laser fluence (please see Figure S6 in Supporting Information).

Finally, we also carried out measurements on CsPbBr_3_ quantum dots and bulk samples under the same excitation laser wavelength set (213, 255, 355, 382, 532, and 775 nm) and experimental conditions. The data is shown in Figure 5. The quantum dots exhibit a clear delayed photoemission component at 355 and 382 nm again, while responses at 213, 255, 532, and 775 nm are predominantly prompt with no resolvable tail. Kinetic analysis of the quantum-dot traces at 355 and 382 nm requires a biexponential model: for 355 nm, τ_fast_ = 130.3 ps and τ_slow_ = 0.989 ns; for 382 nm, τ_fast_ = 220.2 ps and τ_slow_ = 1.046 ns. In bulk CsPbBr_3_, no delayed emission is observed at any wavelength. Because the nanoplatelets and QDs are quantum-confined, whereas the bulk crystal is not, these results indicate that quantum confinement and thus the energy shift of bands enables access to specific conduction bands that support the long-lived highly excited carriers. We note that the absence of delayed photoemission in bulk CsPbBr_3_ does not preclude the existence of such a band: it could be simply because the work function of the band is now too high that electrons cannot be thermally emitted.

**Figure 5:**
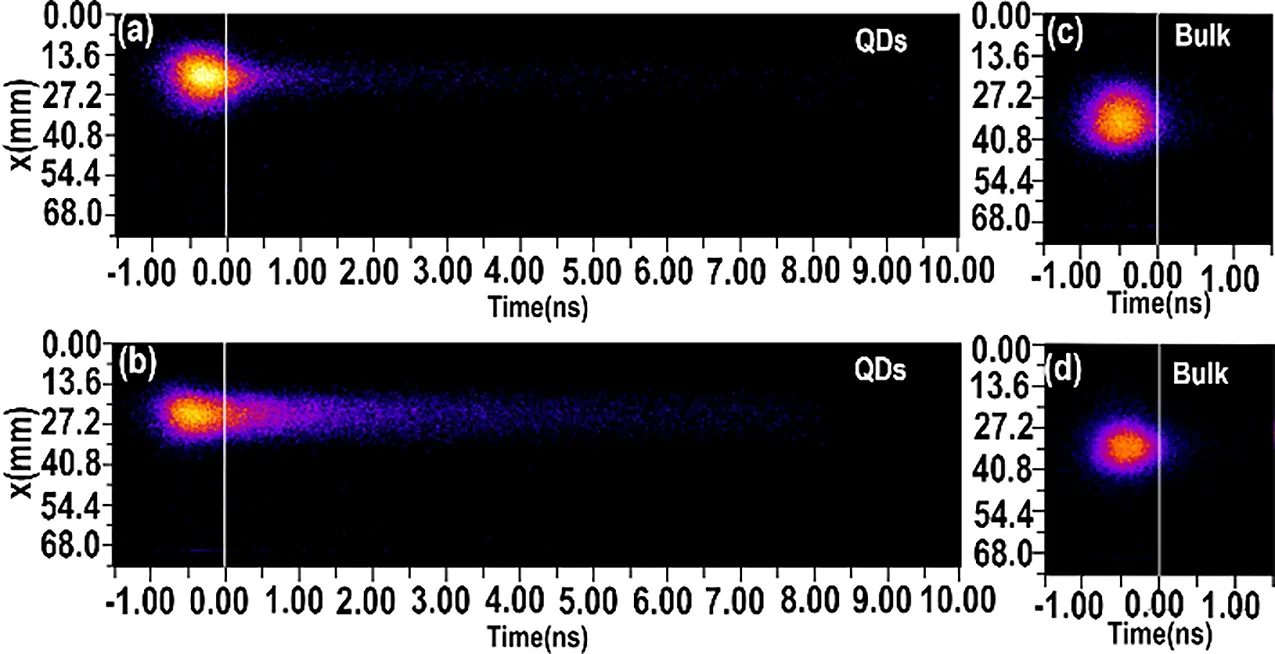
Spatial-TOF distributions of photoelectrons from quantum dots with excitation wavelengths at (a) 355 nm and (b) 382 nm, and those from bulk with (c) 355 nm and (d) 382 nm excitation.

To corroborate these findings and assess the structural origin of the highly excited carrier generation, we performed first-principles calculations of the electronic structure of these 2 ML CsPbBr_3_ NPLs, using density functional theory (DFT) as implemented in the Quantum ESPRESSO package. We built our model based on a published structure from ref.^35^ Here, we replaced the butylammonium in this structure by octylammonium, a ligand used in our experimental work. The geometry relaxations used the SG15 optimized norm-conserving Vanderbilt (ONCV) pseudopotentials,^36–38^ Perdew–Burke–Ernzerhof (PBE) functional,^39^ a kinetic-energy cutoff of 70 Ry, and a **k**-mesh of 4 × 4 × 1, until all forces are below 0.05 eV/Å. The lattice parameters are not relaxed.

Figure 6a shows the optimized structure of the 2 ML CsPbBr_3_ NPL with octylammonium ligands (the “pristine” structure). Its electronic band structure was computed using the PBE functional with spin–orbit coupling (SOC), shown in Figure 6c, where the valence band maximum (VBM) is set to be zero, and the vacuum level is indicated by a blue dashed line. The material possesses a direct band gap at the Γ point of the Brillouin zone. Our calculation confirms the presence of high-energy conduction bands located around the vacuum level. Given the known errors of the PBE functional in predicting band gaps and band edge positions, we proceeded to calculate the electronic structure using the hybrid PBE0 functional^40^ on the uniform **k**-mesh of 4 × 4 × 1, using the Γ-point sampling of the Fock exchange operator. The left column of Figure 6e shows a comparison of the VBM and conduction band minimum (CBM) energies at the Γ point of the Brillouin zone for the pristine structure, calculated from the PBE and PBE0 functionals.

**Figure 6:**
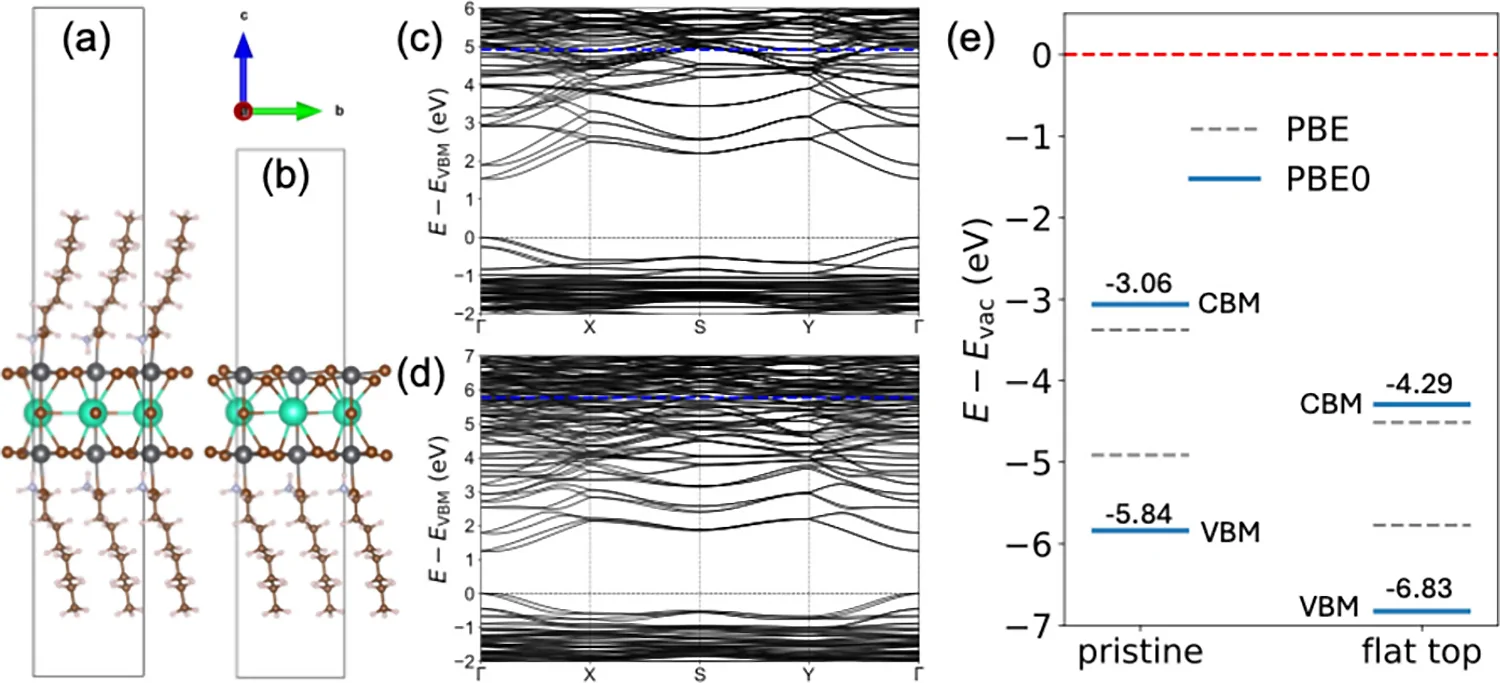
(a) Pristine 2 ML CsPbBr_3_ NPL with octylammonium ligands. (b) 2 ML CsPbBr_3_ NPL with the top organic ligands and Br atoms removed (the “flat top” structure). In panels a and b, the box denotes the periodic simulation cell used in the calculations. The axes compass and labels are also shown. Panels c and d are the PBE band structures of panels a and b, respectively. The VBM energy is set to be zero. (e) VBM and CBM energies at the Γ point of the Brillouin zone for the pristine and “flat top” structures from PBE (dashed gray) and PBE0 (blue) calculations, with the energy of the vacuum level set to be zero.

However, the band energy diagram in the left column of Figure 6e does not explain the experimentally determined mechanism for the generation of highly excited carriers, due to both VBM and CBM energies being too close to vacuum and two-photon excitation leading to direct photoemission. To unveil possible structural origins leading to the highly excited carriers, we considered different types of defects that are common in this type of material,^41^ including both neutral and charged Br, Pb, Cs, H vacancies and their combinations (details are provided in the Supporting Information), and found that none of these structures with point defects possess significantly higher work functions compared to the pristine structure. Therefore, these structures cannot support the photoexcitation mechanism discussed above. Furthermore, we note that the experimentally observed phenomena are similar for both face-down and edge-up samples, and that the edge-up sample features an inorganic, rather than an organic top layer. Based on these considerations, we constructed a “flat top” model shown in Figure 6b, where we removed the organic octylammonium ligands and the neighboring Br atom (for charge neutrality). Its electronic band structure calculated from the PBE functional is shown in Figure 6d, and the comparison of its VBM/CBM energy levels from the PBE/PBE0 functionals is shown in the right column of Figure 6e. We can see that this model significantly lowers the VBM/CBM energies of the pristine structure, due to the substantial change in the surface dipole. The resulting VBM/CBM energies computed from the hybrid PBE0 functional qualitatively support the experimentally determined mechanism for highly excited carrier generation. Therefore, we believe that the model presented in Figure 6b is likely to be the structural origin for the highly excited carrier generation in this type of material.

Based on first-principles calculations, we propose that the formation of an inorganic top contact with vacuum due to the absence of organic ligands provides a material structure capable of trapping photoexcited carriers close enough to the vacuum level that thermal photoemission becomes measurable. We hypothesize the long lifetime of these highly energetic states arises from specific local structures that decouple the electronic and lattice degrees of freedom. This decoupling would slow carrier relaxation processes substantially and enable the observation of long-lived photoemission signals for those charges trapped close to the vacuum level. Two potential candidates capable of producing this effect are local trapping of excited electrons by polaron formation^42^ and long-lived surface excitons.^43^ While the photoemission measurements used in this study average over macroscopic regions of the sample surfaces that inhibit direct probing of these proposed structures, electron transport and vibrational spectroscopic approaches based on atomic force microscopy (AFM) tips could test these hypotheses when coupled with appropriate theoretical approaches. Despite the promise of such studies, they are well beyond the scope of our initial examination of the photoemission behavior here.

In conclusion, we employed TOF three-dimensional electron-momentum imaging under femtosecond/picosecond excitation to probe carrier decay dynamics in undoped 2-ML CsPbBr_3_ nanoplatelets, quantum dots, and bulk crystals. We observe a wavelength-sensitive delayed photoemission channel that is pronounced with 355 and 382 nm but absent at 213, 255, 532, and 775 nm. This indicates the formation of long-lived highly excited carriers by photoexcitation of hot carriers or cooled excitons, which gives rise to the delayed emission. The presence of these long-lived (nanoseconds) highly excited carriers in nanostructures of CsPbBr_3_ is surprising, considering the lower energy carriers cools within a few ps. Nonetheless, these long-lived highly excited carriers have the potential to facilitate hot carrier extraction and support device architectures that target efficiencies beyond the Shockley–Queisser limit, thereby advancing perovskite-based hot-carrier solar cells.

## Supplementary Material



## Notes

Experimental details and sample characterization methods and results (PDF)
