# Supplementary material for: Generating Long-Lived Highly Excited Carriers in Undoped Perovskite Nanostructures by Two-Photon Upconversion
Source: Nano Lett. 2026 Jul 30;26(32):10762–9. doi: 10.1021/acs.nanolett.6c02392 (PMC13495937; doi:10.1021/acs.nanolett.6c02392)
Supplement: Supplementary file 1 [file nl6c02392_si_001.pdf]

Generating Long-Lived Highly-Excited Carriers in Undoped Perovskite Nanostructures by Two-Photon Upconversion

Hackim Musah, Thilina Gamagedara, Estehad Pathan, Amos Afugu, Atish Ghosh, Suk Kyoung Lee, Zhen-Fei Liu, Aaron S. Rury, and Wen Li*

Department of Chemistry, Wayne State University, Detroit, MI, 48202, USA

*wli@chem.wayne.edu

1. Materials Synthesis and Sample Fabrication

Synthetic Methods

We used ligand-assisted reprecipitation method at room temperature to synthesize cesium lead bromide ($CsPbBr_3$) nanoplatelets. We made 0.4 M $PbBr_2$ solution by dissolving 294 mg of $PbBr_2$ in 2 mL of N,N-dimethyl formamide (DMF). We added 1.25 mL of octadecene, 0.125 mL oleic acid, and 0.125 mL of oleyl amine in a glass vial to form the ligand solution. We formed cesium oleate precursor by adding 350 mg cesium carbonate to a solution mixture containing 20 mL of octadecene and 1.25 mL of oleic acid. We heated the solution for 90 minutes at 135 ˚C under constant stirring and purging with purified Argon. We then added 300 μL of the 0.4 $PbBr_2$ solution and 20 μL of HBr (48% by volume) to the ligand solution. Last, we added 200 μL the precursor solution to the ligand solution swiftly and quenched with 5 mL of acetone after 15 s. The resulting solution was centrifuged at 4000 rpm for 10 minutes and the resulting precipitate was dispersed in 1.5 mL of either hexane or toluene.

Oriented Film Preparation

Thin films of $CsPbBr_3$ nanoplatelets were formed by dropping solutions made following centrifugation onto silicon substrates (MTI Corp., >10 MΩ resistivity). As shown by previous scanning transmission electron microscopy measurements,[1] films cast from $CsPbBr_3$ nanoplatelet dispersions in toluene formed with nano-particles oriented with their confined direction parallel to the plane of the substrate, which we call edge-up (EU). In contrast, films cast from $CsPbBr_3$ nanoplatelet dispersions in hexane formed with constituent nano-particles oriented with their confined axes perpendicular to the substrate plane, which we call face-down (FD).

2. Materials Characterization

    2.1 Nanoplatelet sample characterization

UV–Visible absorbance

Absorption spectra were recorded using a Jasco V-770 UV–Vis–NIR spectrophotometer. Nanoplatelet and quantum dot dispersions were transferred into quartz cuvettes for solution-phase absorbance measurements. The absorption spectra of the 2ML $CsPbBr_3$ nanoplatelets dispersed in hexane and toluene are presented in Figure 1c of the main text. The optical characterization of the $CsPbBr_3$ quantum dots, including UV–Vis absorption and photoluminescence spectra, is shown in Figure S3.

Reflection and Photoluminescence Spectroscopy

Reflection spectra were measured using a custom-built spectrometer with a deuterium light source (Thorlabs SLS204), fiber-coupled to an achromatic collimator (Thorlabs ZC618FC-A), and detected using a fiber-coupled spectrometer (Ocean Optics OceanFX). Samples were prepared by drop-casting nanoplatelet dispersions onto a Si wafer. The orientations of the sample and spectrometer fiber inlet were controlled with independent, motorized rotation stages (Thorlabs PRM1Z8) with 0.1˚ precision.

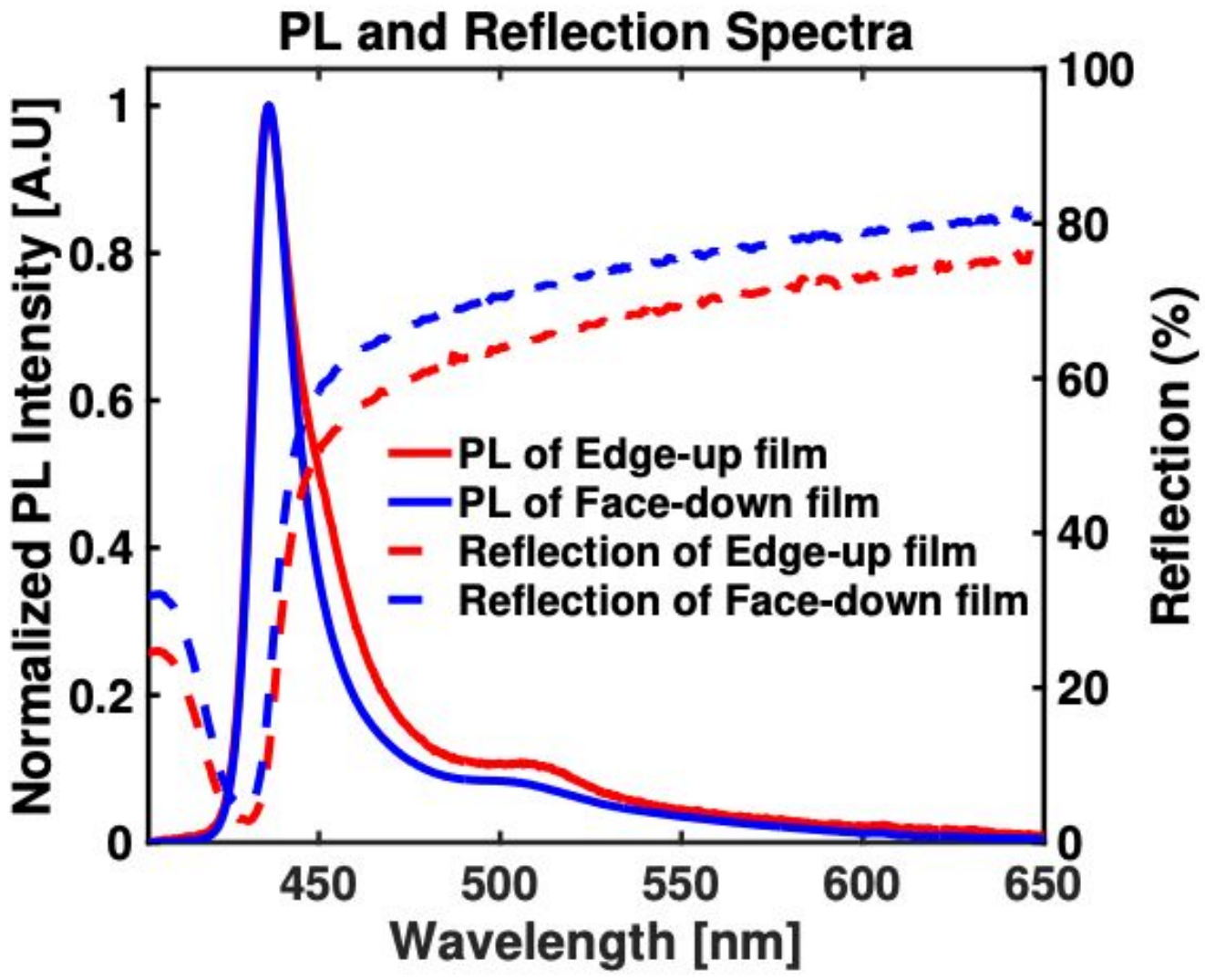

Figure S1. *Comparisons between the reflection (dashed) and photoluminescence (solid) spectra of CsPbBr3 nanoplatelet edge-up (red) and face-down (blue) films.*

Steady-state photoluminescence (PL) spectra were obtained using a custom-built, free space apparatus with a 405 nm continuous-wave laser as the excitation source. The emitted light was collected in a backscattering geometry and analyzed with a 1500 mm spectrograph (Andor Shamrock) equipped with a CCD detector (Andor Newton). For solution measurements, nanoplatelet dispersions were placed in a quartz cuvette. Thin films were prepared by drop-casting the dispersion onto a clean fused silica substrate. The excitation power was set to 1 mW for solution measurements and 2 μW for thin films to avoid photo-induced sample damage. Figure S1 shows that the spectra of thin films cast from hexane and toluene dispersions do not differ significantly. Both reflection spectra are characterized by a dip at 430 nm due to absorption by the excitons in the $CsPbBr_3$ nanoplatelets. Additionally, peaks at 437 nm appear most prominently in the PL spectra of films cast from both solvents. These similarities indicate that the $CsPbBr_3$ nanoplatelets in each film have the same electronic structures.

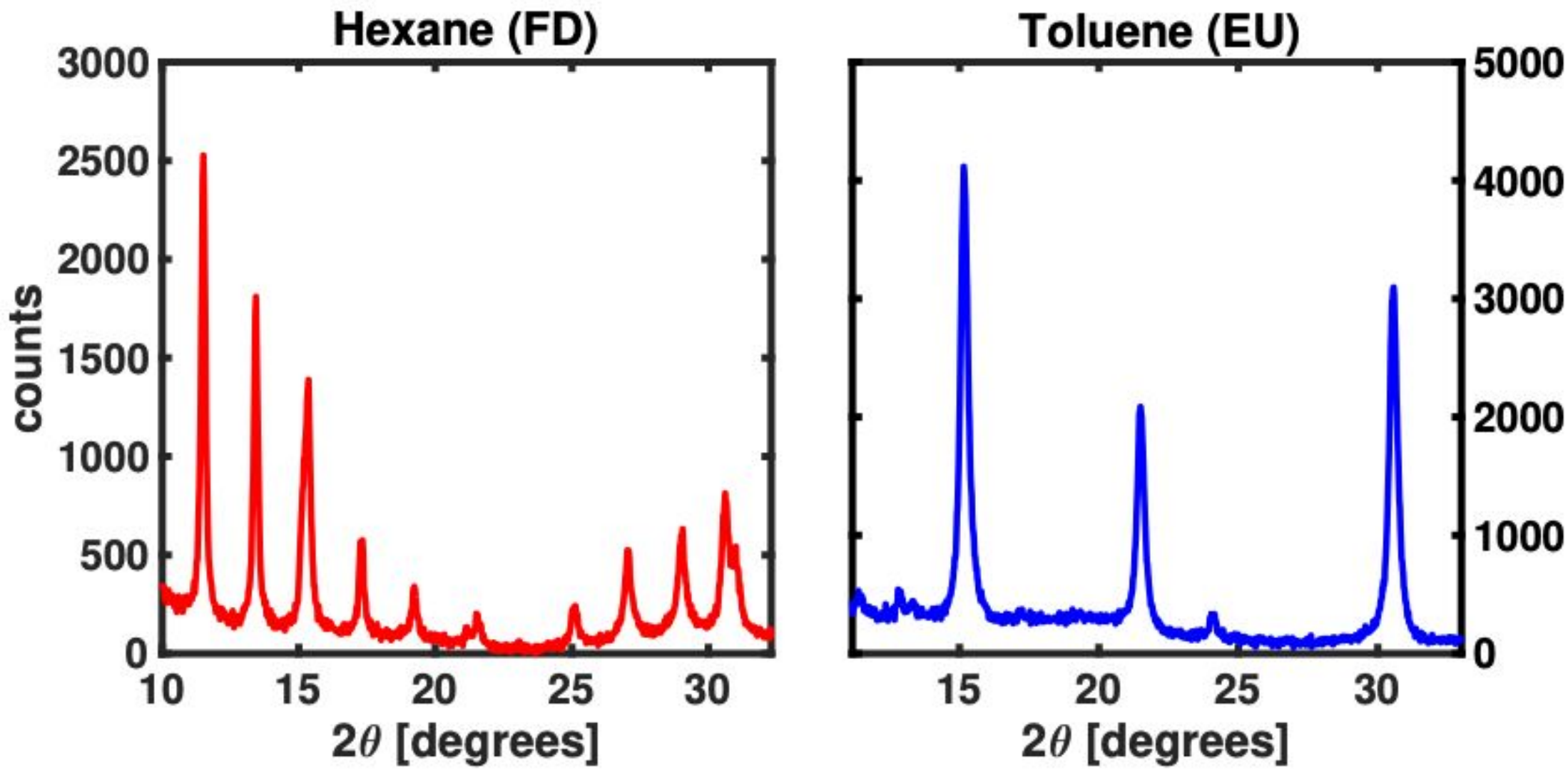

Figure S2. *X-ray diffraction patterns scattered by $CsPbBr_3$ nanoplatelet films cast from dispersions in 1.5 mL of hexane (left) and toluene (right), which show the distinct peaks associated with face-down (FD) and edge-up (EU) orientations, respectively, of the underlying nanoparticles.*

X-ray Diffraction

Samples for X-ray diffraction (XRD) measurements were prepared by drop-casting nanoplatelet dispersions onto a silicon wafer. XRD patterns were recorded using a Bruker D2 Phaser diffractometer operating at 30 kV and 10 mA, equipped with a Cu radiation source and a LYNXEYE™ detector. All measurements were carried out at 300 K. Figure S2 shows the XRD patterns scattered from $CsPbBr_3$ nanoplatelet films cast from hexane and toluene dispersions. Comparison of these patterns indicates important differences related to the orientations of nano-particles in films cast from dispersions using different solvents. For the patterns scattered by the sample cast from the hexane dispersion, we find closely spaced peaks centered around $2\theta = 10^\circ$ and $2\theta = 31^\circ$, which are consistent with reflections due to in-plane Pb-Pb and Pb-Br distances. The modulations that ride on these reflections result from interference between the X-rays that scattered from $CsPbBr_3$ nanoplatelets that form superlattices in the face-down samples, as reported previously. In contrast, we observe only three significant peaks at $2\theta = 15^\circ$, $2\theta = 22.5^\circ$, and $2\theta = 31^\circ$in the XRD pattern scattered by our $CsPbBr_3$ nanoplatelet film cast from the toluene dispersion. These peaks stem from the out-of-plane Pb-Pb, Br-Br, and Pb-Br distances and confirm the edge-up orientation of the $CsPbBr_3$ nanoplatelet in samples formed from toluene dispersions. The association with distinct orientations has been corroborated by scanning transmission electron microscopy previously.[1]

High-Angle Annular Dark-field Scanning Transmission Electron Microscopy (HAADF-STEM)

Scanning transmission electron microscopy (STEM) images were collected using a Talos F200X operated at an accelerating voltage of 200 kV in high-angle annular dark-field (HAADF) mode. Samples were prepared by drop-casting a dilute nanoplatelet colloidal solution onto carbon-coated copper grids (Ted Pella, 400 mesh). After drying under ambient conditions, the grids were mounted on a single-tilt holder for imaging. Representative HAADF-STEM images comparing the morphology of the 2ML

$CsPbBr_3$ nanoplatelet films prepared from hexane and toluene dispersions are shown in Figure 1(a) and (b) of the main text.

Additionally, the same Talos F200X microscope was used in conventional TEM mode to characterize the size of the $CsPbBr_3$ quantum dots (QDs). As shown in Figure S3a, representative TEM measurements give particle dimensions of approximately **5.8–6.9 nm**, with an average size of **6.35 nm**. This value is slightly smaller than the reported exciton Bohr diameter of $CsPbBr_3$, approximately **7 nm**, indicating that the quantum dots are sufficiently small to exhibit quantum confinement effects. These measurements provide further structural support for assigning the observed delayed emission to quantum-confined $CsPbBr_3$ quantum dots.

2.2 Quantum dot sample characterization

The properties of the $CsPbBr_3$ quantum dots were also characterized using TEM, UV-Vis absorption (AB) and photoluminescence (PL) spectroscopy. The optical spectra were normalized for comparison, as shown in Figure S3b. The absorption spectrum shows a peak near 500 nm, while the photoluminescence spectrum peaks at 509 nm, consistent with band-edge optical transitions in quantum-confined $CsPbBr_3$ quantum dots. This is in good agreement with the TEM image showing the typical size of the quantum dot to be ~6 nm (Fig. S3a).

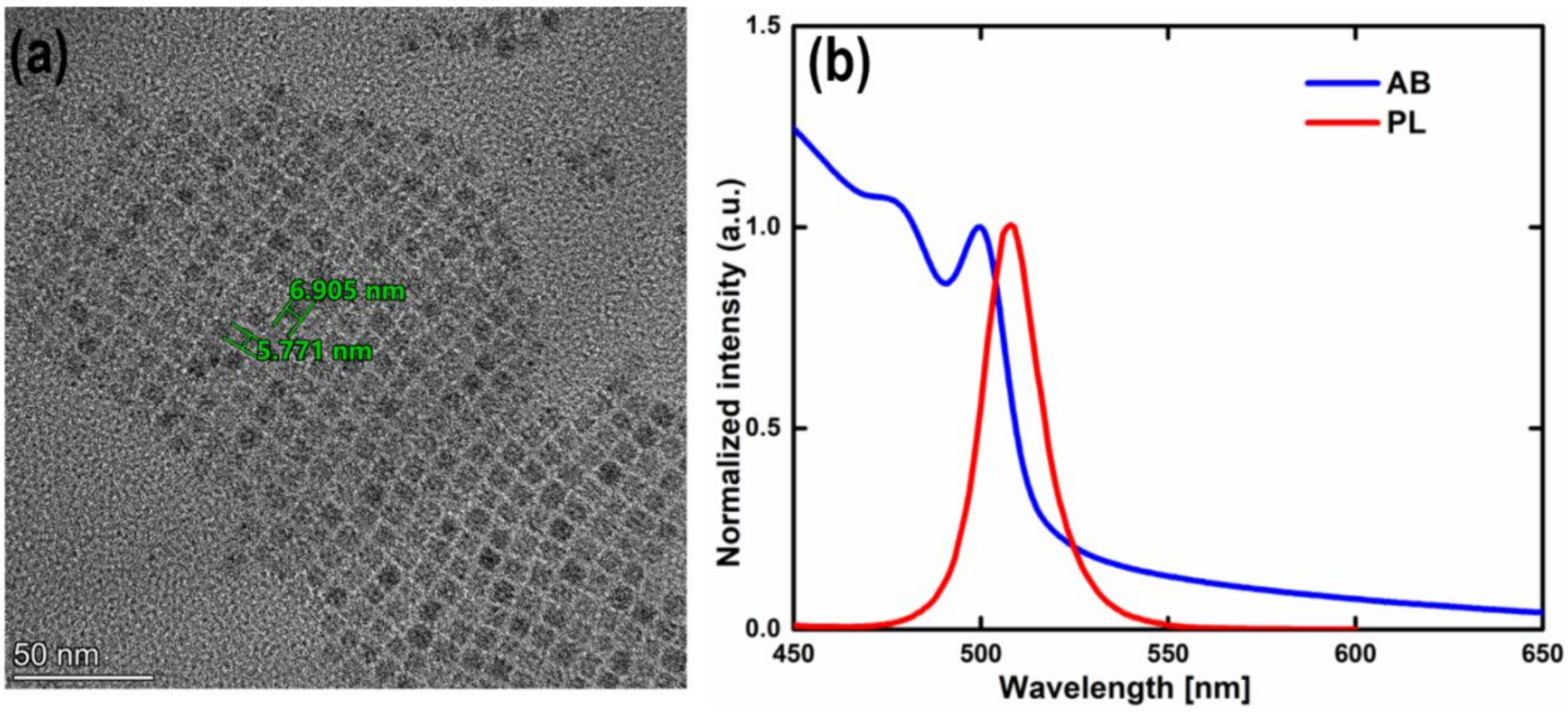

***Figure S3**. Characterization of $CsPbBr_3$ quantum dots.*
(a) TEM image and (b) Absorption (AB) and Photoluminescence (PL) spectra of the $CsPbBr_3$ QDs.

2.3 X-ray photoelectron spectroscopy on 2-ML $CsPbBr_3$ nanoplatelets

XPS measurements were performed using a Thermo Fisher Scientific Nexsa spectrometer equipped with a monochromatic Al Kα X-ray source. The analysis area was 400 µm in diameter. High-resolution spectra were acquired for the Cs 3d, Pb 4f, Br 3d, O 1s, N 1s, and C 1s core levels using a pass energy of 50 eV. Charge neutralization was applied during data acquisition using a flood gun to compensate for surface charging. All binding energies were calibrated using the C 1s peak at 284.8 eV.

The XPS spectra were processed using MATLAB. A linear baseline (straight line) was defined by selecting intensity values at the low- and high-binding-energy edges of

each spectral region and fitting these points with a linear function. This baseline was then subtracted from the raw spectra before performing peak fitting.

The baseline-corrected spectra were fitted using Gaussian line shapes in the MATLAB Curve Fitting Toolbox. For core levels showing spin–orbit coupling (such as Pb 4f, Cs 3d, and Br 3d), two Gaussian components were used to represent the expected doublets, modeled as:

$$I(x) = a_1 \exp\left(-\left(\frac{x-b_1}{c_1}\right)^2\right) + a_2 \exp\left(-\left(\frac{x-b_2}{c_2}\right)^2\right) + dx + e \quad \textbf{Eq. (S1)}$$

where x represents the binding energy, a_2 the peak amplitude, b the peak position, and c the width of each Gaussian component, while $dx + e$ accounts for any residual

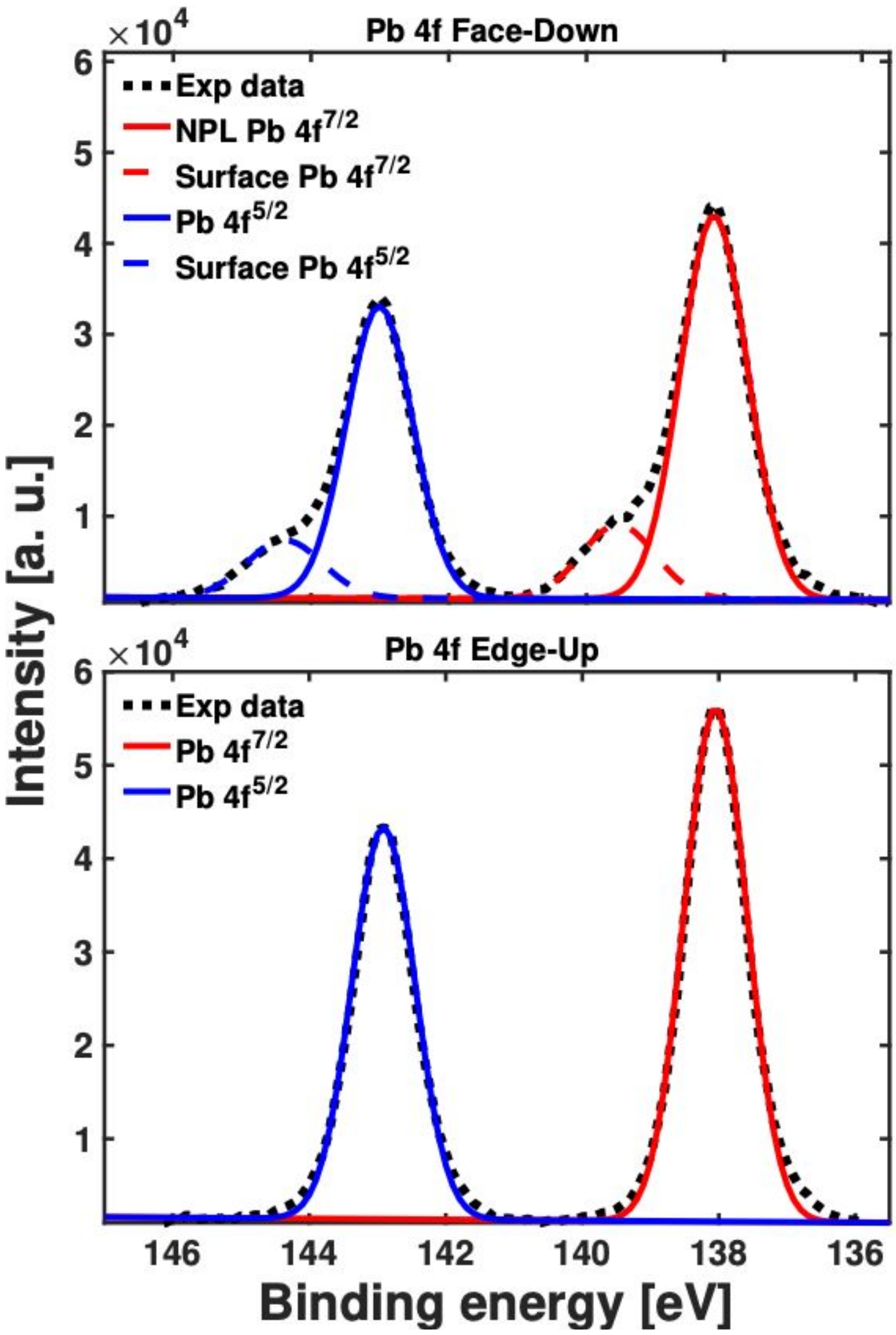

Figure S4. *Comparisons between the measured XPS peaks corresponding to Pb 4f core electrons (dotted black) and Gaussian fits of the $4f^{7/2}$ (solid red) and $4f^{5/2}$ (solid blue) for face-down (top panel) and edge-up (bottom panel) $CsPbBr_3$ nanoplatelet films. The top panel also shows comparisons between the high energy shoulders in experimental results and Gaussian fits in the $4f^{7/2}$ (dashed red) and $4f^{5/2}$ (dashed blue) regions, which we assign to surface states resulting from poor Pb passivation.*

linear background. When additional spectral features or further peak splitting were observed, extra Gaussian components were included to achieve better fits.

We summarize our XPS results on dropcast films on Si substrate in Table S1. This table shows that we observe an overall decrease in the position of the peaks corresponding to Cs, Br and Pb when comparing the samples cast from dispersions in hexane to those cast from toluene mixtures. These shifts vary from 0.03 eV to 0.1 eV depending on the peak. We also observe the appearance of distinct shoulders on the high energy sides of the Pb 4f peaks, which are shifted by nearly 1.5 eV for both *J* values.

Table S1. XPS results summary

Solvent	Cs (eV)	Cs (eV)	Br (eV)	Br (eV)	Pb (eV)	Pb (eV)	Pb (eV)	Pb (eV)
Hexane	724.06±0.01	737.02±0.02	67.99±0.02	69.09±0.03	138.14±0.02	139.54±0.02	143.01±0.02	144.39±0.11
Toluene	724.03±0.01	736.99±0.02	67.96±0.02	69.05±0.03	138.05±0.02	-	142.91±0.02	-

Previous studies show that surface degradation of the inorganic perovskite structure to non-terminated Pb cations can manifest as blue-shifts to 4f XPS peaks.[2] These peaks coincide more closely with those of orthorhombic $PbBr_2$.[2] As seen in Fig. S6, our XPS measurements show that doublets appear in vicinity of the spin-orbit split $4f^{7/2}$ and $4f^{5/2}$ peaks in NPL film samples drop cast from hexane solutions. We propose that the higher energy shoulders of each doublet appear in the spectrum due to unterminated Pb cations like those predicted by our DFT calculations to shift the surface levels downward relative to the pristine material. We do not see the same doublets in the case of NPL films drop cast from toluene solutions, which would be consistent with our observing smaller thermionic emission signals in our time-of-flight measurements. The background free nature of the time-resolved photoemission measurements makes detecting those signals easier than in the XPS experiments where other, more pristine regions of the sample overwhelm the results.

These features in the XPS spectrum of our FD samples of the $CsPbBr_3$ nanoplatelets differ from those attributed to surface oxidation. In the case of exposing the surface to water and inducing the appearance of the O 1s peak in XPS spectra, Ha et al. observed a small (0.02 eV) shift of the Pb peaks. This significantly smaller shift than the value we find in Figure S4 suggests that more significant perturbations than surface oxidation must play a role in our samples.[3] These perturbations could include modifications of the surface structures, like those consistent with the predictions of our DFT model.

3. 3D momentum imaging of photoemission from edge-up 2-ML $CsPbBr_3$ nanoplatelets

By repeating the photoelectron measurements on edge-up 2-ML $CsPbBr_3$ nanoplatelets under the same conditions as the face-down sample, we obtained the X–Z maps shown in Figure S5. Only 355 nm and 382 nm excitation produce a pronounced delayed population arriving after t_0(positive time) and persisting for ~8–

10 ns, which dominates the integrated signal. In contrast, excitation at 532, 775, 213, and 255 nm yields predominantly prompt emission with at most a weak tail that decays within ≲0.25 ns, i.e., no resolvable delayed component within the measurement window.

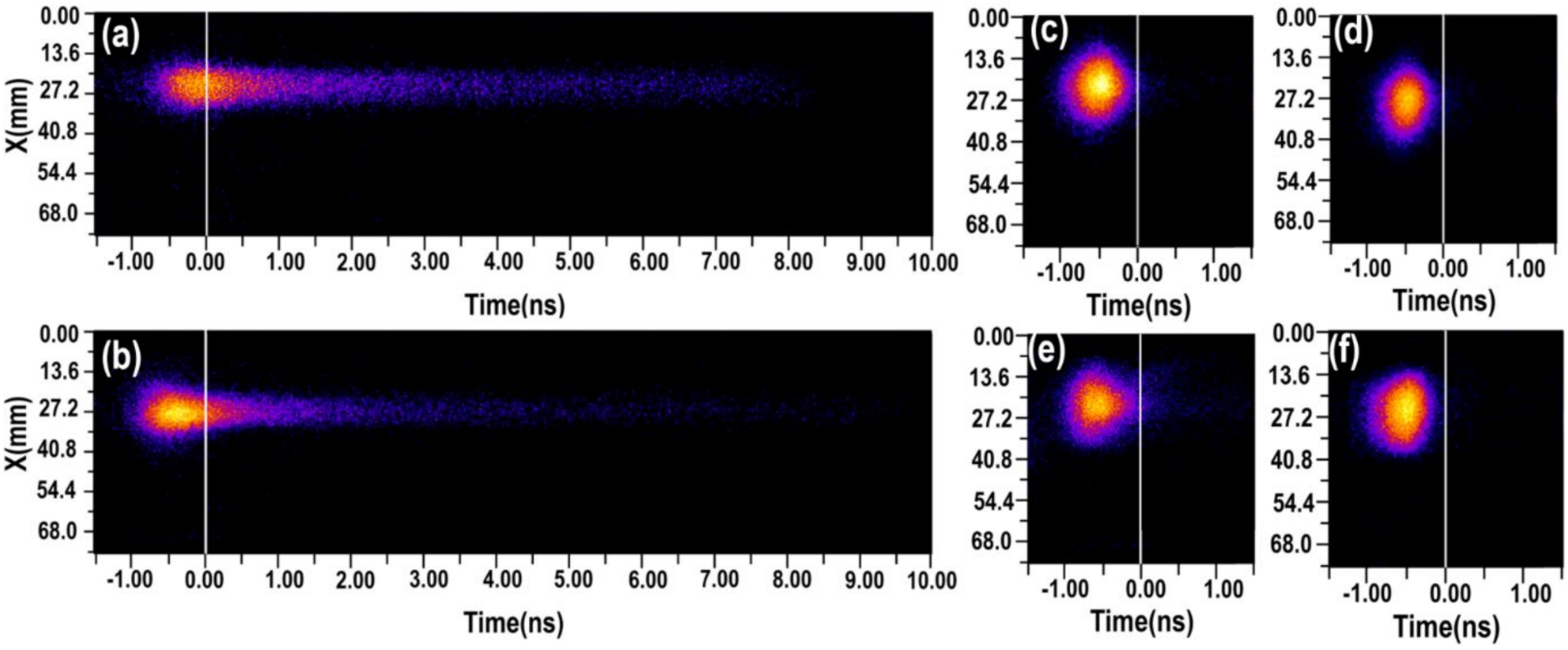

Figure S5. *Spatial-TOF distributions of photoelectrons emitted from edge-up 2-ML $CsPbBr_3$ nanoplatelets. The horizontal axis is the TOF axis while X is a transverse spatial coordinate. Time zero (p_z = 0), as specified in the main text, is indicated by the vertical white line. Excitation wavelengths: (a) 355 nm, (b) 382 nm, (c) 532 nm, (d) 775 nm, (e) 213 nm, and (f) 255 nm.*

4. Fluence dependent TOF decays, power law dependence, pulse duration characterization and carrier cooling dynamics

We probed the effect of excitation density by measuring TOF decays as a function of fluence. Within the examined range, the fitted lifetimes remained unchanged to within

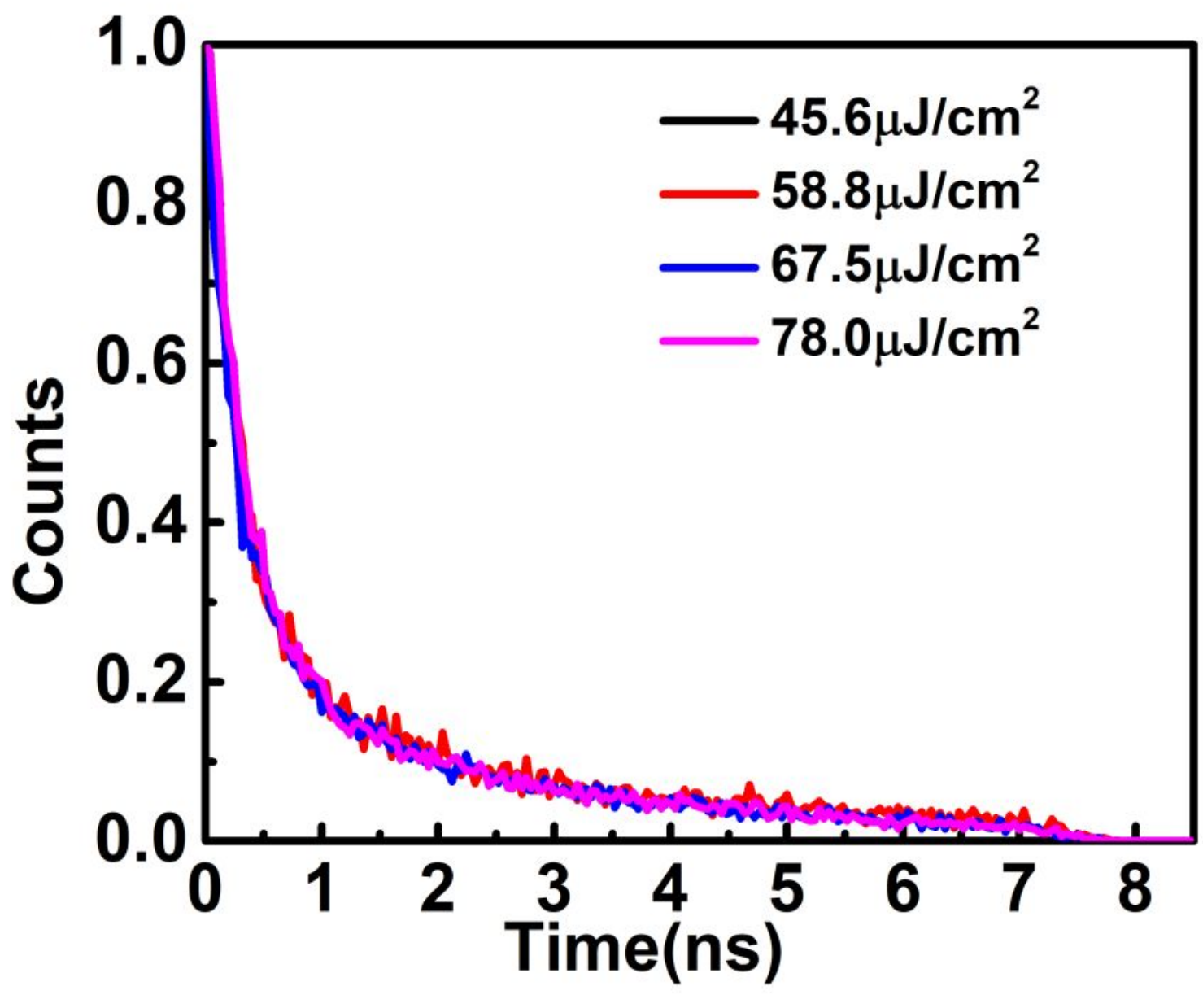

Figure S6. *Fluence-dependent measurement on the 2ML $CsPbBr_3$ Nanoplatelets*

uncertainty, as shown in Figure S6. This agreement indicates that the delayed component is effectively independent of pump fluence under our conditions.

To evaluate the excitation intensity dependence, the integrated electron yield was analyzed as a function of excitation fluence for both **355 nm** and **382 nm** excitation. In the log–log representation shown in Figure S7, the total electron yield follows power-law slopes of **1.91** for 355 nm excitation and **1.83** for 382 nm excitation, indicating a near-quadratic dependence at both wavelengths. This behavior is consistent with a predominantly two-photon-driven excitation process under the conditions studied.

From the same analysis, the apparent threshold fluences were estimated to be **37.49μJcm⁻²** for 355 nm excitation and **2.29μJcm⁻²** for 382 nm excitation. The lower threshold observed at 382 nm suggests that delayed emission is initiated more efficiently under this excitation condition. However, the comparable power-law exponents at both wavelengths indicate that, once above threshold, the excitation process remains strongly nonlinear for both excitation wavelengths. For comparison, the fluence dependence under 775 nm excitation was also analyzed and is shown in Figure S7b. The 775 nm data exhibit a higher-order power-law slope of 3.74, consistent with a multiphoton process involving approximately four photons. This comparison further distinguishes the 355 and 382 nm excitation pathways from the higher-order 775 nm excitation process.

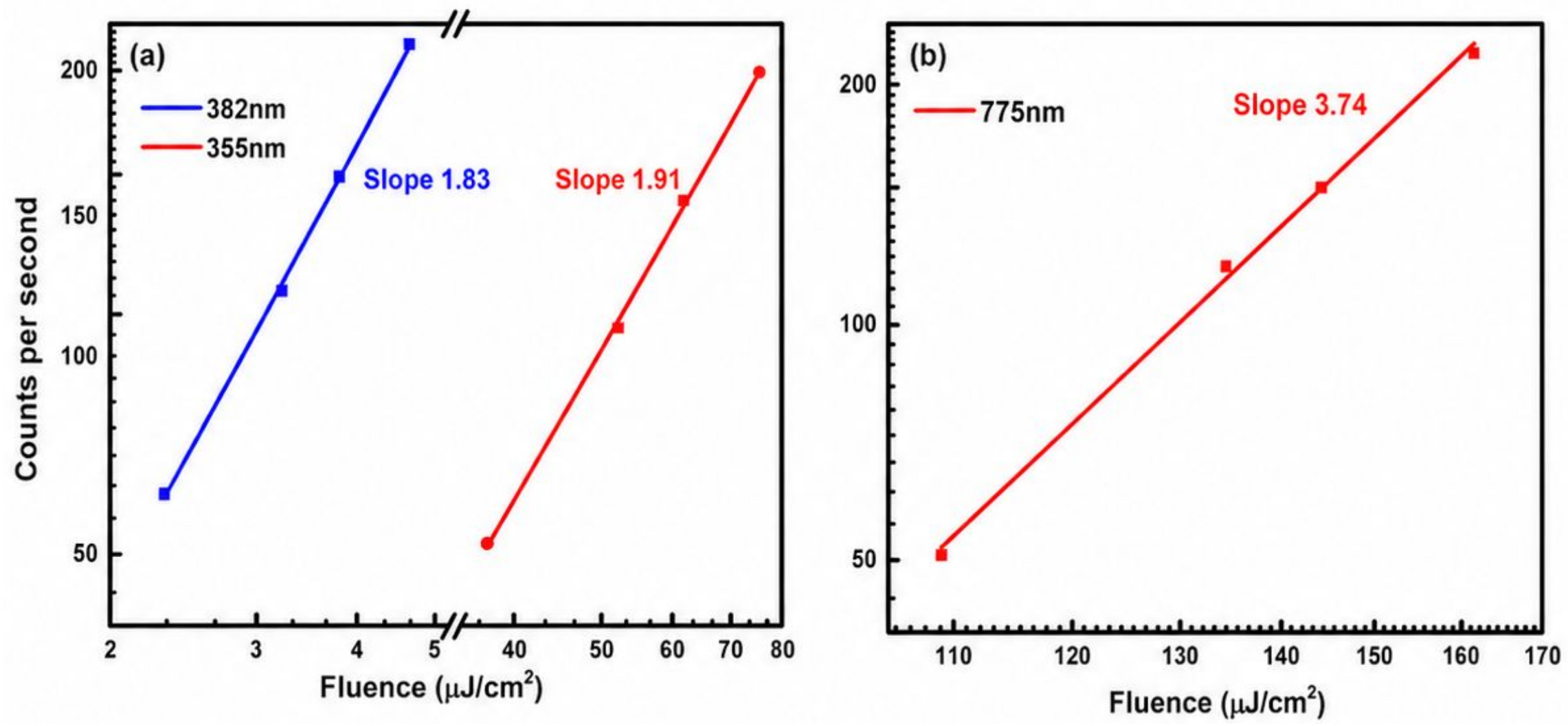

Figure S7. *Power-law dependence of electron counts per second(cts/s) on excitation fluence for (a) 355 and 382 nm excitation and (b) 775 nm excitation.*

To compare the excitation efficiency at different wavelengths, the photon fluence was first calculated at the condition that produced an electron yield rate of 0.02 electrons per pulse, which is the instrument threshold for detecting electrons. The number of incident photons required to generate one detected electron was then calculated considering the size of the NPL and the detector efficiency of 50%. The calculated values are summarized in **Table S2**. The data shows that the femtosecond 382 nm laser is the most efficient to produce thermionic emission, while 775nm is the least

efficient due to its non-resonance and high-photon order nature. It is interesting to note 355 nm picosecond pulse is less efficient than femtosecond 382 nm pulse. Considering both photons can access the conduction band with one photon absorption, this suggests the pulse duration plays an important role in the dynamics. The efficiency difference can be explained by the energy of the highly excited states by 382 nm being higher than that by 355 nm, which leads to a high emission rate.

Wavelength(nm)	Photon Fluence @ 0.02 electron/pulse (photons/cm^2/pulse)	#Photons needed for 1 electron emission
355	1.78×10^{13}	16000
382	2.23×10^{12}	2000
532	2.66×10^{13}	24000
775	4.20×10^{14}	378000

Table S2. Wavelength dependent photon fluence for producing detectable electron counts and calculated number of photons needed for producing a single photoelectron.

To characterize the temporal resolution of the instrument, we performed third harmonic generation using 775 nm and 382nm in a thin BBO crystal. In the beam path, we added additional glass window to match the group velocity dispersion that is introduced by the glass window of the vacuum chamber. The delay-dependent third-harmonic intensity (Fig. S8) was then fit with a Gaussian function to determine the effective 382 nm–775 nm cross-correlation. This measurement yielded a cross-correlation FWHM of 157.76 fs. We also measured the 775 nm autocorrelation, which gave a Gaussian FWHM of 89.1 fs.

Assuming Gaussian pulse profiles, the single-pulse duration of the 775 nm beam was determined to be 63.0 fs. The measured 382 nm–775 nm cross-correlation width contains contributions from both the 382 nm and 775 nm pulses. After accounting for the independently measured 775 nm pulse duration, the 382 nm pulse duration under the experimental beam-path conditions was estimated to be 144.6 fs. This value represents the effective 382 nm pulse duration at the sample position under our experimental optical-path conditions.

Next, we measured the hot-carrier cooling dynamics in the $CsPbBr_3$ nanoplatelets using a time-resolved two-photon photoemission (2PPE) pump–probe experiment. In this measurement, a 382 nm pulse was used to excite the sample, while a delayed 775 nm pulse served as the probe to photoemit electrons from the transient excited-state population. The pump–probe delay was varied using an optical delay stage, and the integrated photoelectron yield was recorded as a function of delay time. The obtained transient response is shown in Figure S8 and was analyzed using a single-exponential decay convolved with a Gaussian instrument response function (IRF) **(Equation S2)**. The Gaussian standard deviation from the fit was σ_g=75.5 ± 3.19 fs, corresponding to an effective temporal full width at half maximum of $\mathrm{FWHM} = 2\sqrt{2\ln 2}\,\sigma_g$= 176.97 ± 7.50 fs.

$$y(t) = C + \frac{A}{2}\mathbf{exp}\left(\frac{\sigma^2}{2\tau^2} - \frac{t-t_0}{\tau}\right)\mathbf{erfc}\left(\frac{\sigma^2/\tau-(t-t_0)}{\sqrt{2}\,\sigma}\right) \quad \textbf{Eq. (S2)}$$

where t represents the femtosecond pump-probe delay, y(t) is the measured count rate, C is the baseline offset, and A is the amplitude of the transient signal. The parameter τ represents the single-exponential decay lifetime, which is 2049.22 ± 94.96 fs, t_0 is the fitted time-zero position, and sigma is the Gaussian width of the instrument response function. The complementary error function, erfc, arises from the analytical convolution of the exponential decay with the Gaussian response.

The effective IRF obtained from the 2PPE fit is in reasonable agreement with the independently measured third-harmonic 382 nm–775 nm cross-correlation FWHM of 157.76 fs, as shown in Figure S8. The agreement between these two measurements confirms that the pulse duration of 382 nm is ~145 fs under our experimental optical-path conditions. More importantly, the much slower cooling rate at 2 ps shows that the pulse duration of the 382nm can indeed produce highly excited states before significantly cooling can take place. This corroborates the discussion on mechanism 2 and 3 in the main text.

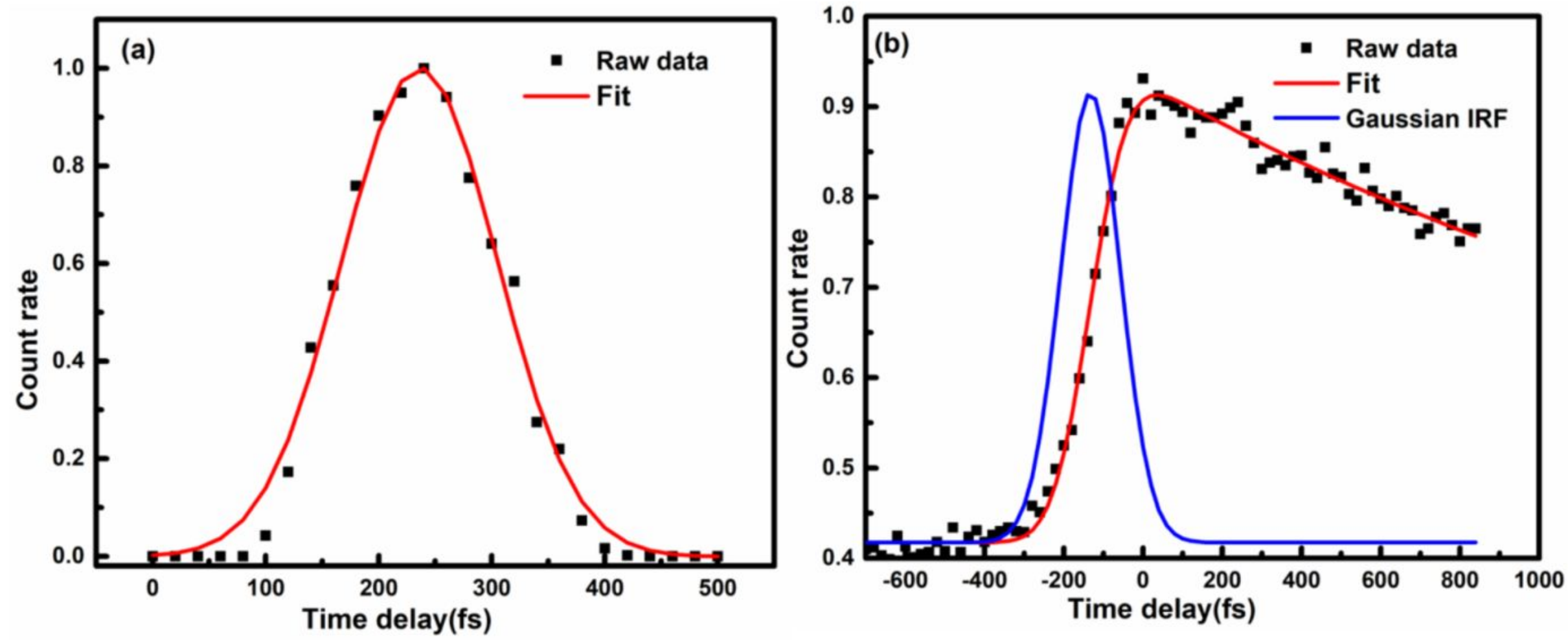

Figure S8. *Temporal characterization of the excitation pulses. (a) Third-harmonic cross-correlation of the 382 nm and 775 nm beams. (b) 2PPE transient response fit with a single-exponential decay convolved with a Gaussian IRF.*

5. Surface-defect models and electronic structure calculations

To further examine how surface defects and incomplete surface passivation may influence the electronic structure, a series of $CsPbBr_3$ defect models were constructed and evaluated using DFT calculations. These models include different atom-removal configurations from the exposed top surface and from the inorganic plane, allowing comparison between surface-localized and lattice-associated defects.

The optimized defect structures are shown in Figure S9, and the corresponding PBE band structures are presented in Figure S10. Comparison of these systems provides insight into how missing Br, Pb, Cs, or neighboring ligand-related atoms influence the electronic states relative to the vacuum level. These calculations support the

discussion that surface defects and incomplete passivation can alter the local electronic environment of $CsPbBr_3$ and may contribute to the prolonged hot-carrier lifetime associated with the observed delayed emission.

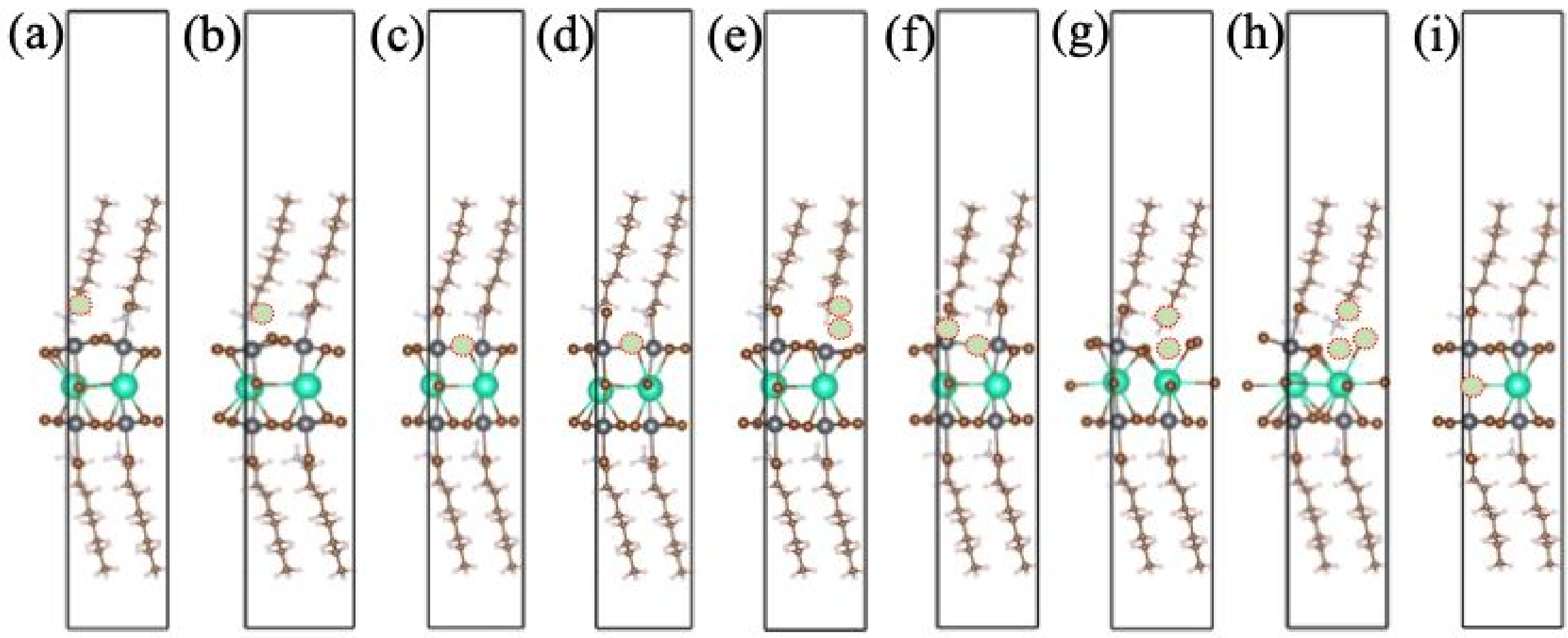

Figure S9. *Different defect systems studied in this work, in addition to the "flat-top" structure in the main text. Red circles with filled green color denote the positions of the atoms removed from the pristine structure. (a) A neutral Br atom removed from the top. (b) A Br^- removed from the top. (c) A neutral Br atom removed from the plane. (d) A Br^- removed from the plane. (e) A neutral Br atom and a neutral H atom from the neighboring NH_4 removed from the top. (f) A neutral Br atom and a neutral H atom from the neighboring NH_4 removed from the plane. (g) A neutral Br atom removed from the top, together with a neutral Pb atom.* (h) A neutral Br atom removed from the top, with another neutral Br atom removed from the plane and a neutral Pb atom removed. (i) A neutral Cs atom removed.

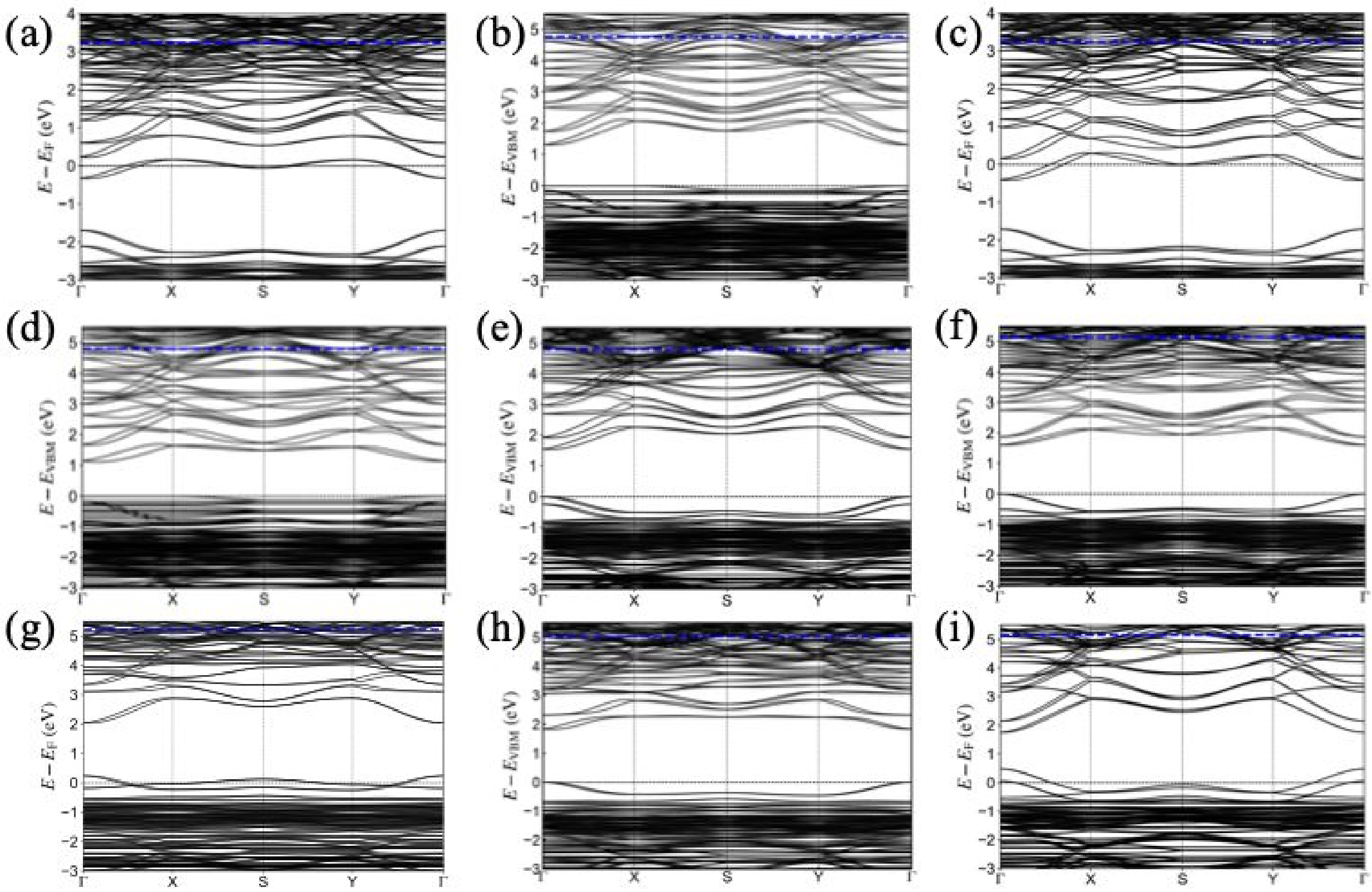

Figure S10. PBE band structures of different defect systems studied in this work. Their structures are shown in ***Figure S9****. In all plots, the dashed blue line denotes the vacuum level.*

Table S3. Gaps of the pristine and a few defect systems. "Vac." stands for the vacuum level. PBE results are reported without the parentheses. PBE0 results are reported within parentheses. The Figure number is listed for reference. All values are in eV.

	Figure	Vac.-CBM	CBM-VBM	Vac.-VBM
Pristine	5(a)	3.37 (3.06)	1.54 (2.78)	4.91 (5.84)
Flat-top	5(b)	4.52 (4.29)	1.25 (2.54)	5.77 (6.83)
(1) a neutral Br atom removed from the top	S2(a)	3.57	1.37	4.94
(2) Br^- removed from the top	S2(b)	3.45	1.31	4.76
(3) a neutral Br atom removed from the plane	S2(c)	3.65 (4.45)	1.31 (1.49)	4.96 (5.94)
(4) Br^- removed from lateral plane	S2(d)	3.65 (3.29)	1.15 (2.62)	4.80 (5.91)
(5) (1) + a neutral H from OA ligand removed	S2(e)	3.25	1.55	4.80
(6) (3) + a neutral H from OA ligand removed	S2(f)	3.53	1.61	5.14
(7) (1) + a neutral Pb atom removed	S2(g)	3.18	1.79	4.97
(8) (1) + (3) + a neutral Pb atom removed	S2(h)	3.19	1.84	5.03
(9) a neutral Cs atom removed	S2(i)	3.40	1.27	4.67

*For some defect systems [(1)(3)(7)(9) above], the electronic structure is technically a metal (i.e., the Fermi level lies within a band). For these systems, in the numbers reported in this table, we still consider the VBM (CBM) as the highest (lowest) band below (above) the large apparent gap that is similar to that in the pristine structure, for a fair comparison across all systems.
**For the two charged systems [(2)(4) above], the vacuum level is determined by using the vacuum level of the corresponding neutral system and aligning the lowest occupied (core) orbitals between the charged system and the corresponding neutral system, i.e., E(vacuum, charged) = E(vacuum, neutral) + [E(lowest occupied orbital, charged) – E(lowest occupied orbital, neutral)].

Atomic Coordinates
Pristine structure

```
CELL_PARAMETERS angstrom
8.2868003845      0.0000000000      0.0000000000
0.0000000000      8.1518001556      0.0000000000
0.0000000000      0.0000000000     50.0000000000
ATOMIC_POSITIONS angstrom
Pb     2.1450287095    4.6289788925    22.7006599738
Pb     6.1417437746    0.5528697105    16.5837949142
Pb     6.1417705000    0.5530793484    22.7006605217
Pb     2.1450566548    4.6287697263    16.5837947716
Cs     1.8223549021    0.3352451955    19.6422559564
Cs     6.4644453145    4.4111451069    19.6422558951
Br     2.1416786140    4.6153926610    19.6422314994
Br     6.1451217986    0.5394923528    19.6422313461
Br     0.4539499560    2.1200767263    16.7025503783
Br     0.4539962233    2.1202231553    22.5819603187
Br     3.5006681131    7.2994829692    22.6811371222
Br     4.7860498377    3.2232988389    16.6034699736
Br     4.7861318942    3.2235828493    22.6811370488
Br     3.5007502378    7.2991993567    16.6034699066
Br     1.8717229332    4.8048396124    25.7285180175
Br     6.4150625960    0.7286974944    13.5559833138
Br     6.4150771991    0.7289395115    25.7285176866
Br     1.8717378184    4.8045975517    13.5559828359
Br    -0.4539964344    6.1961232496    22.5819601184
Br    -0.4539498819    6.1959767106    16.7025504041
N      6.9386590619    4.0017359268    25.1992335689
N      6.9388510619    4.0013716268    14.0854881854
N      1.3479490183   -0.0745284520    14.0854885637
N      1.3481414848   -0.0741635569    25.1992325790
H      6.8161302038    3.0005611299    25.4708022479
H      6.8162136199    3.0002830301    13.8140706410
H      7.9362941357    4.2682611515    25.3565840833
H      0.3504439610    0.1920349885    13.9282157942
H      0.3505058383    0.1923614759    25.3565843601
H      7.9363558719    4.2679349604    13.9282158835
H      6.7495843742    4.0566373064    24.1858507057
```

H	6.7497664244	4.0562889912	15.0991404001
H	4.9858436757	4.5164613928	25.7651729805
H	3.3008784340	0.4401796325	13.5198063684
H	3.3009563397	0.4405613914	25.7651727676
H	4.9859216746	4.5160795625	13.5198061700
H	6.0726849688	5.8909167259	25.4830966011
H	2.2140908119	1.8146438568	13.8019454384
H	2.2141153094	1.8150167927	25.4830965350
H	6.0727092177	5.8905438501	13.8019452708
H	7.3249027465	5.3991239586	27.5653277137
H	0.9619212125	1.3230673705	11.7196710288
H	0.9618972127	1.3232240527	27.5653276920
H	7.3248788255	5.3989672876	11.7196711030
H	6.3547281602	3.9467124195	27.8498887548
H	6.3547901661	3.9465252628	11.4350357242
H	5.1686055935	6.7735858714	27.6607713026
H	3.1183069255	2.6974006549	11.6243918184
H	3.1181944930	2.6976861159	27.6607717232
H	5.1684931434	6.7733008757	11.6243924430
H	4.2904122206	5.2982839583	28.0472888304
H	3.9963884613	1.2220706376	11.2377460803
H	3.9963873857	1.2223844962	28.0472898115
H	4.2904115276	5.2979704796	11.2377471040
H	5.9041347291	5.0674186177	30.0912641443
H	2.3826813648	0.9915036681	9.1937407509
H	2.3826665010	0.9915200204	30.0912662975
H	5.9041196825	5.0674023288	9.1937427735
H	6.3797607879	6.7317510290	29.7680971972
H	1.9071418645	2.6558279974	9.5170683788
H	1.9070396269	2.6558520587	29.7680992487
H	6.3796578917	6.7317278325	9.5170694457
H	1.9320100234	-0.1293748332	11.4350358212
H	1.9320720327	-0.1291872042	27.8498888286
H	1.4705864916	-1.0756172452	13.8140703886
H	1.4706696996	-1.0753389161	25.4708026333
H	1.5370335707	-0.0196109051	15.0991406761
H	1.5372152815	-0.0192623139	24.1858517832
H	3.9443655485	7.4240962506	9.3333727983
H	3.5098278401	5.7522324681	9.0295660171
H	5.0775227337	5.8460927396	6.9612115147
H	5.1684440006	7.5822671800	7.2171437806
H	2.6439661640	7.7098506021	7.0399294939
H	2.5941192572	5.9787359432	6.7502144843
H	3.9168403372	6.2568770923	4.6350159010
H	2.4749340614	7.2904379670	4.5871220622
H	4.0634501307	7.9939699020	4.9305254368
H	4.7769733934	1.6763314509	9.0295637765
H	4.3424377481	3.3481985862	9.3333828006
H	3.2092219268	1.7702065385	6.9611978828
H	3.1183533411	3.5063978632	7.2171511068

H	5.6926940079	1.9028326252	6.7502137025
H	5.6428556850	3.6339541608	7.0399251047
H	5.8118672082	3.2145381384	4.5871182440
H	4.3699603269	2.1809768028	4.6350158351
H	4.2233530825	3.9180699748	4.9305309288
H	4.7769106027	1.6764362862	30.2555734674
H	4.3423282369	3.3483097230	29.9518502948
H	3.2091039763	1.7701601709	32.3239243103
H	3.1182098312	3.5063643042	32.0680589893
H	5.6925382476	1.9028079714	32.5350129578
H	5.6426980300	3.6339230088	32.2453135382
H	4.3697682188	2.1809570192	34.6501877662
H	5.8116776838	3.2145045269	34.6981130430
H	4.2231782370	3.9180477585	34.3546716651
H	3.9444759782	7.4242078413	29.9518588977
H	3.5098936581	5.7523371304	30.2555762160
H	5.0776575568	5.8460493527	32.3239072787
H	5.1686007426	7.5822367750	32.0680649523
H	2.6440699089	7.7098111067	32.2452970536
H	2.5942742183	5.9786819092	32.5350189141
H	2.4751230094	7.2904017366	34.6981041539
H	3.9170359896	6.2568544431	34.6501959429
H	4.0636258069	7.9939490313	34.3546745855
C	5.9991914442	4.8974303922	25.9413599301
C	2.2875926411	0.8212233406	13.3436634638
C	2.2876086439	0.8215305884	25.9413611896
C	5.9992074768	4.8971232363	13.3436638361
C	6.3231421705	4.9637873639	27.4264376634
C	1.9636163231	0.8876493958	11.8585299889
C	1.9636584050	0.8878881927	27.4264390453
C	6.3231839327	4.9635491256	11.8585307734
C	5.2668462297	5.7964731136	28.1578687419
C	3.0200160840	1.7203448765	11.1272079576
C	3.0199539895	1.7205738377	28.1578706940
C	5.2667841316	5.7962448632	11.1272091848
C	5.5541896695	6.0122172640	29.6453136293
C	2.7326635126	1.9362468677	9.6397813147
C	2.7326119089	1.9363194179	29.6453167080
C	5.5541369109	6.0121451997	9.6397832807
C	4.3143864099	6.4910742822	8.8804116765
C	4.5252933779	6.7025156677	7.3797566159
C	3.2164171594	6.8764364445	6.6037431842
C	3.4252047491	7.1177078937	5.1084245063
C	3.9724145921	2.4151784605	8.8804131680
C	3.7615013590	2.6266345730	7.3797582352
C	5.0703949969	2.8005365211	6.6037430453
C	4.8616000990	3.0418081034	5.1084251393
C	3.9723171725	2.4152571637	30.4047513241
C	3.7613783712	2.6266118855	31.9054165155
C	5.0702403044	2.8005151242	32.6814707582

C	4.8614227421	3.0417810740	34.1767850568
C	4.3144881663	6.4911532799	30.4047529154
C	4.5254282013	6.7024977901	31.9054165096
C	3.2165677774	6.8764007820	32.6814710105
C	3.4253835254	7.1176766229	34.1767858704

Flat-top structure

CELL_PARAMETERS angstrom

8.2868003845	0.0000000000	0.0000000000
0.0000000000	8.1518001556	0.0000000000
0.0000000000	0.0000000000	40.0000000000

ATOMIC_POSITIONS angstrom

Pb	2.1494579646	4.5834106097	22.8413228728
Pb	6.1640395694	0.5127847360	16.8521084313
Pb	6.1373510368	0.5075103435	22.8413161345
Pb	2.1227691156	4.5886887523	16.8521029326
Cs	1.9748782969	0.2176498664	20.0881148784
Cs	6.3119286343	4.2935540119	20.0881164911
Br	2.5252615420	4.6017045199	19.9624397665
Br	5.7615478390	0.5258089589	19.9624349597
Br	0.4710393243	2.0507887127	17.0471075068
Br	0.2636590898	2.2886738638	22.4567350595
Br	3.6254155489	7.1459368695	23.2010369372
Br	4.7873987568	3.1783629035	16.7837458324
Br	4.6613927643	3.0700371285	23.2010394030
Br	3.4994084422	7.2542629271	16.7837382807
Br	6.4616877026	0.6952907551	13.8419462172
Br	1.8251055341	4.7711905231	13.8419468563
Br	-0.2636503044	6.3645730965	22.4567364225
Br	-0.4710237533	6.1266970559	17.0471113323
N	6.8557761134	4.0163511516	14.2729358528
N	1.4310801460	-0.0595736956	14.2729854580
H	6.7432094537	3.0170319980	13.9957598838
H	0.4262805620	0.1993534486	14.1638023457
H	7.8604888760	4.2752271308	14.1638056881
H	6.6118434679	4.0584077371	15.2782775654
H	3.3513526981	0.4984209605	13.6428900115
H	4.9354539734	4.5743351223	13.6428963227
H	2.2405670314	1.8455278541	13.9410364145
H	6.0462485483	5.9214270872	13.9410452127
H	0.9397736097	1.2845691676	11.8885300991
H	7.3470272036	5.3604664378	11.8885273856
H	6.3237799721	3.9474966812	11.5944819823
H	3.0124561558	2.7528848364	11.7158953768
H	5.2743639502	6.8287919903	11.7158942327
H	3.9684798824	1.3172219141	11.3803433599
H	4.3183132763	5.3931412743	11.3803756009
H	2.3743664524	0.9396594557	9.3295955147
H	5.9123720754	5.0155152720	9.3296100450

H	1.8160422090	2.5856803142	9.6069207734
H	6.4707574970	6.6615194754	9.6069088232
H	1.9630149392	-0.1284033523	11.5944659090
H	1.5435729727	-1.0588628944	13.9957478841
H	1.6749411374	-0.0174918257	15.2782375792
H	4.0567428525	7.4553234141	9.4492327056
H	3.5628273707	5.7960001922	9.1506845366
H	5.1216427714	5.8348575456	7.0742003547
H	5.2462652954	7.5723499457	7.3048833772
H	2.7187471865	7.7412489513	7.1414289118
H	2.6344955963	6.0009647198	6.9273692577
H	4.0025159343	6.2120571845	4.7909572906
H	2.4807473343	7.1153503979	4.7061312638
H	4.0064356861	7.9705310314	4.9911405410
H	4.7239337325	1.7200516386	9.1506584377
H	4.2300973872	3.3793853386	9.4492582993
H	3.1650602863	1.7590692691	7.0741914124
H	3.0406266697	3.4965701225	7.3049148234
H	5.6522115934	1.9249056095	6.9273206524
H	5.5681721268	3.6651903942	7.1414377029
H	5.8060494020	3.0393162128	4.7061076651
H	4.2841560204	2.1362358622	4.7909484277
H	4.2804912311	3.8946964306	4.9911805673
C	2.3239020181	0.8497040800	13.4898497093
C	5.9628487195	4.9255676338	13.4898607174
C	1.9606340094	0.8915646690	12.0108988289
C	6.3262003901	4.9674884637	12.0109004626
C	2.9670446497	1.7574134443	11.2488650758
C	5.3197362552	5.8333004005	11.2488754793
C	2.6750570160	1.9116757004	9.7532162221
C	5.6117276412	5.9875547000	9.7532281463
C	4.3889802121	6.5092254398	8.9941315120
C	4.5895767401	6.7060399328	7.4893695607
C	3.2691980679	6.8800316755	6.7309640385
C	3.4467651431	7.0551464806	5.2233208326
C	3.8978196942	2.4333253461	8.9941244303
C	3.6972191064	2.6301884361	7.4893623792
C	5.0176219476	2.8040475490	6.7309542074
C	4.8400232953	2.9792356521	5.2233211743

REFERENCES

(1) Krajewska, C. J.; Kaplan, A. E. K.; Kick, M.; Berkinsky, D. B.; Zhu, H.; Sverko, T.; Van Voorhis, T.; Bawendi, M. G. Controlled assembly and anomalous thermal expansion of ultrathin cesium lead bromide nanoplatelets. *Nano Lett.* **2023**, *23* (6), 2148-2157. DOI: 10.1021/acs.nanolett.2c04526.
(2) Zhidkov, I. S.; Boukhvalov, D. W.; Akbulatov, A. F.; Frolova, L. A.; Finkelstein, L. D.; Kukharenko, A. I.; Cholakh, S. O.; Chueh, C.-C.; Troshin, P. A.; Kurmaev, E. Z. XPS spectra as a tool for studying photochemical and thermal degradation in APbX3

hybrid halide perovskites. *Nano Energy* **2021**, *79* (105421), 105421. DOI: 10.1016/j.nanoen.2020.105421.
(3) Ha, S. K.; Mauck, C. M.; Tisdale, W. A. Toward stable deep-blue luminescent colloidal lead Halide perovskite nanoplatelets: Systematic photostability investigation. *Chem. Mater.* **2019**, *31* (7), 2486-2496. DOI: 10.1021/acs.chemmater.8b05310.
